# Supplementary material for: Chimeric GPCRs mimic distinct signaling pathways and modulate microglia responses
Source: Nat Commun. 2022 Aug 15;13:4728. doi: 10.1038/s41467-022-32390-1 (PMC9378622; doi:10.1038/s41467-022-32390-1)
Supplement: Supplementary file 1 — Supplementary Information [file 41467_2022_32390_MOESM1_ESM.pdf]

Supplementary Figure S1

Confirming alignment-identified domains with TMHMM predictions

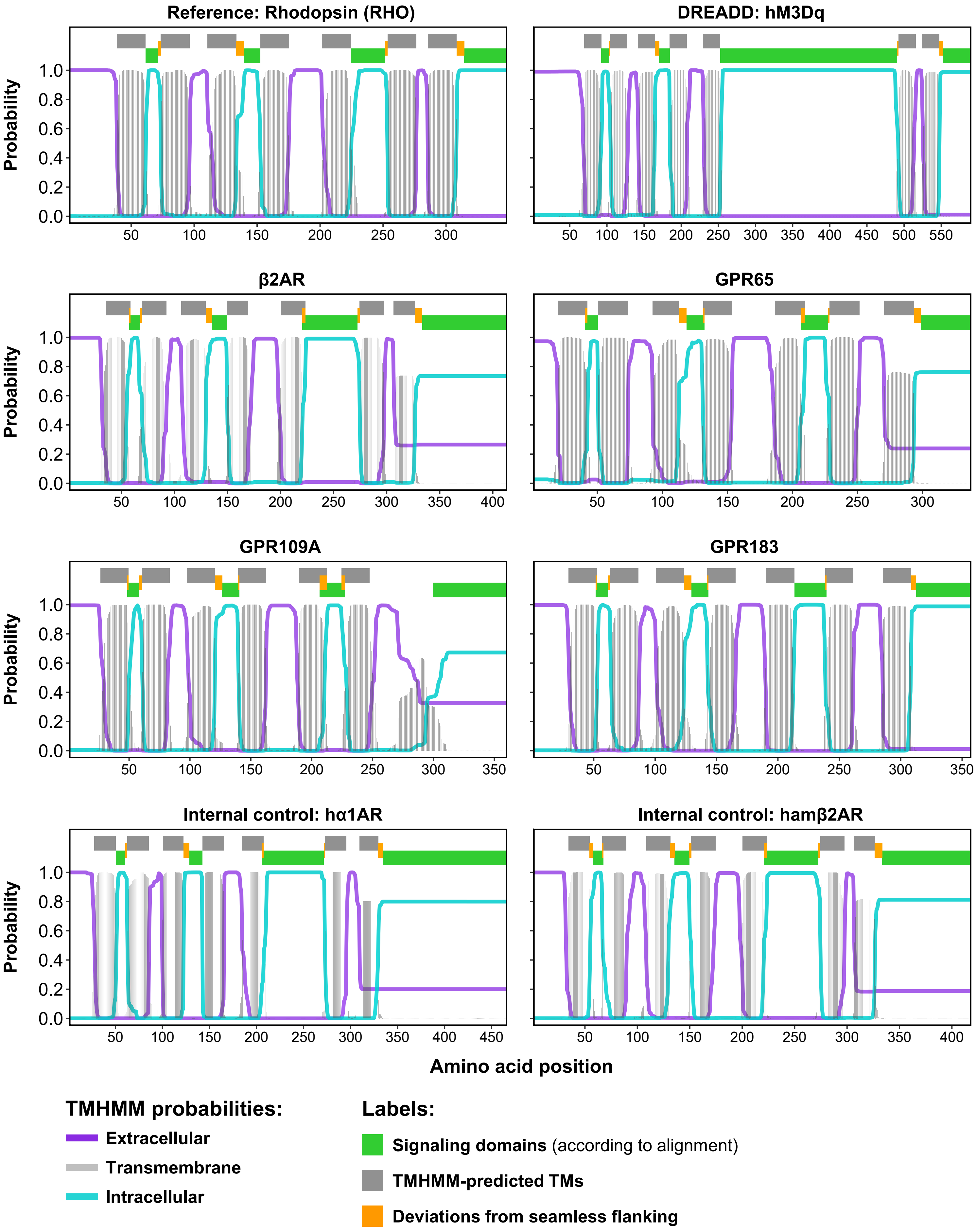

Supplementary Figure S1: TMHMM-predictions support multiple protein sequence alignment accuracy.

TMHMM probability plots for rhodopsin (RHO), hM3Dq, four representative GPCRs-of-interest, human α1-adrenergic receptor (hα1AR), and hamster β2-adrenergic receptor (hamβ2AR). TMHMM reports the probability that a GPCR sequence is located in the extracellular (purple line), transmembrane (light grey line), or intracellular (light turquoise line) space. The top of each plot shows the results from multiple protein sequence alignment from N- to C-terminus. Alignment-identified intracellular signaling domains (green bars) were tightly flanked by TMHMM-predicted transmembrane domains (TMs; dark grey bars). Deviations from seamless flanking (orange bars) were minimal and comparable to deviations within the alignment reference (rhodopsin) and internal controls (hα1AR, hamβ2AR). Source data are provided as a Source Data file.

Supplementary Figure S2

Introducing N-terminal modifications

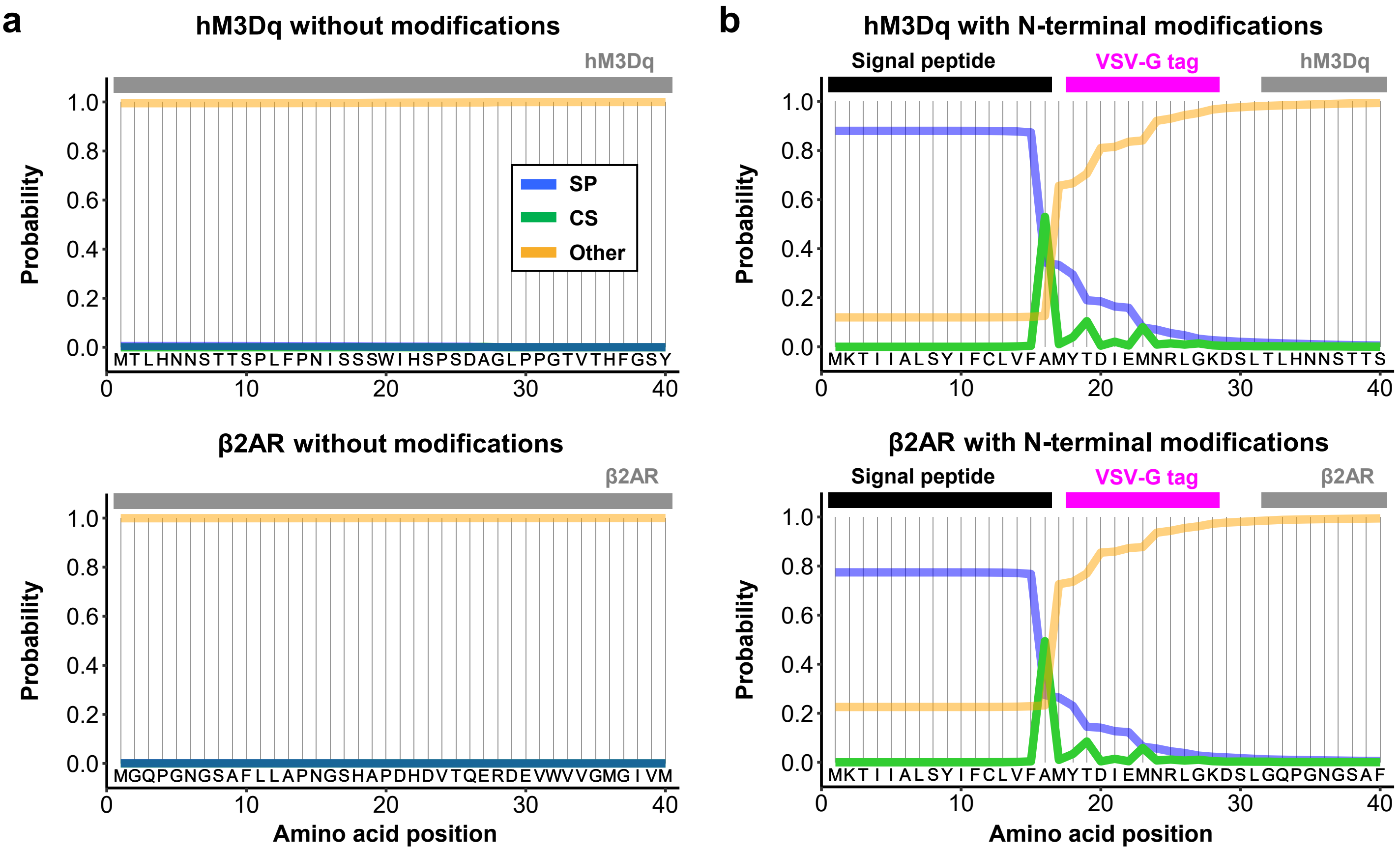

Confirming surface expression

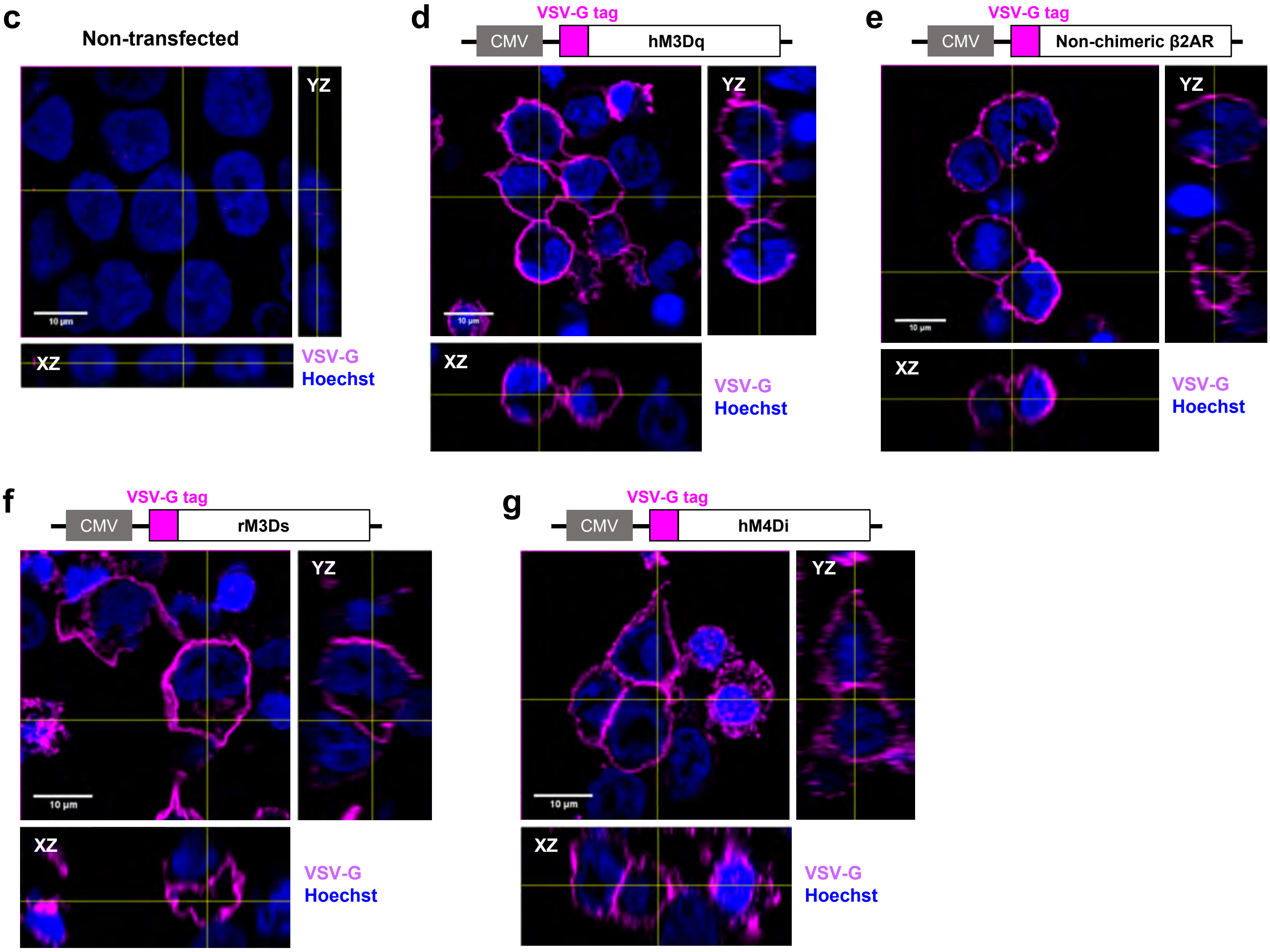

Supplementary Figure S2: N-terminal modifications of DREADDs and β2AR are compatible with cell surface expression.

a-b: SignalP probability plots for hM3Dq (top) and β2AR (below) without any modification (**a**) or with signal peptide and VSV-G epitope tag (**b**). Blue line (SP): probability that sequence belongs to a signal peptide. Green line (CS): probability of signal peptide cleavage. Orange line (Other): probability that sequence does not contain a signal peptide. Grey bar represents protein sequence of either hM3Dq or β2AR. Source data are provided as a Source Data file. **c-e:** Orthogonal views of HEK cells immunostained for VSV-G tag under non-permeabilizing conditions in non-transfected cells (**c**), and cells transfected with hM3Dq (**d**), non-chimeric β2AR (**e**), rM3Ds (**f**), or hM4Di (**g**). Magenta: VSV-G tag. Blue: nuclear staining with Hoechst. CMV, human cytomegalovirus promoter.

Supplementary Figure S3

a *Endogenous β2AR contributes only partially to the response of β2AR-transfected HEK cells*

Dose-dependent contribution to cAMP synthesis evaluated with two β2AR ligands

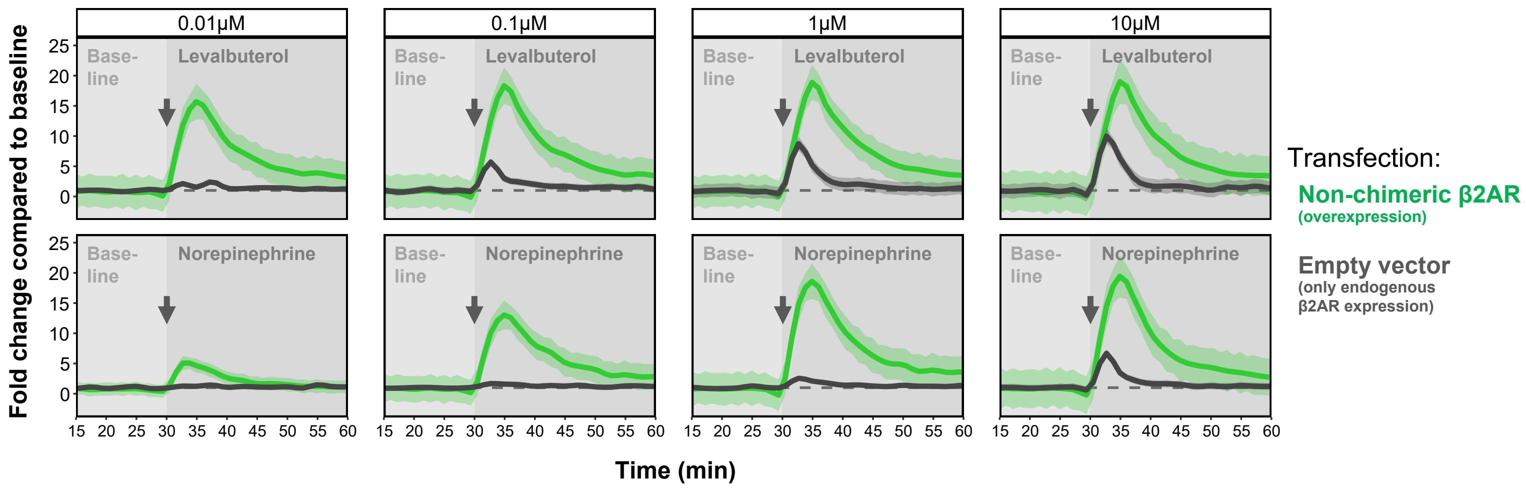

b *The DREADD-β2AR and rM3Ds response is saturated at 10µM CNO (measured through cAMP increase)*

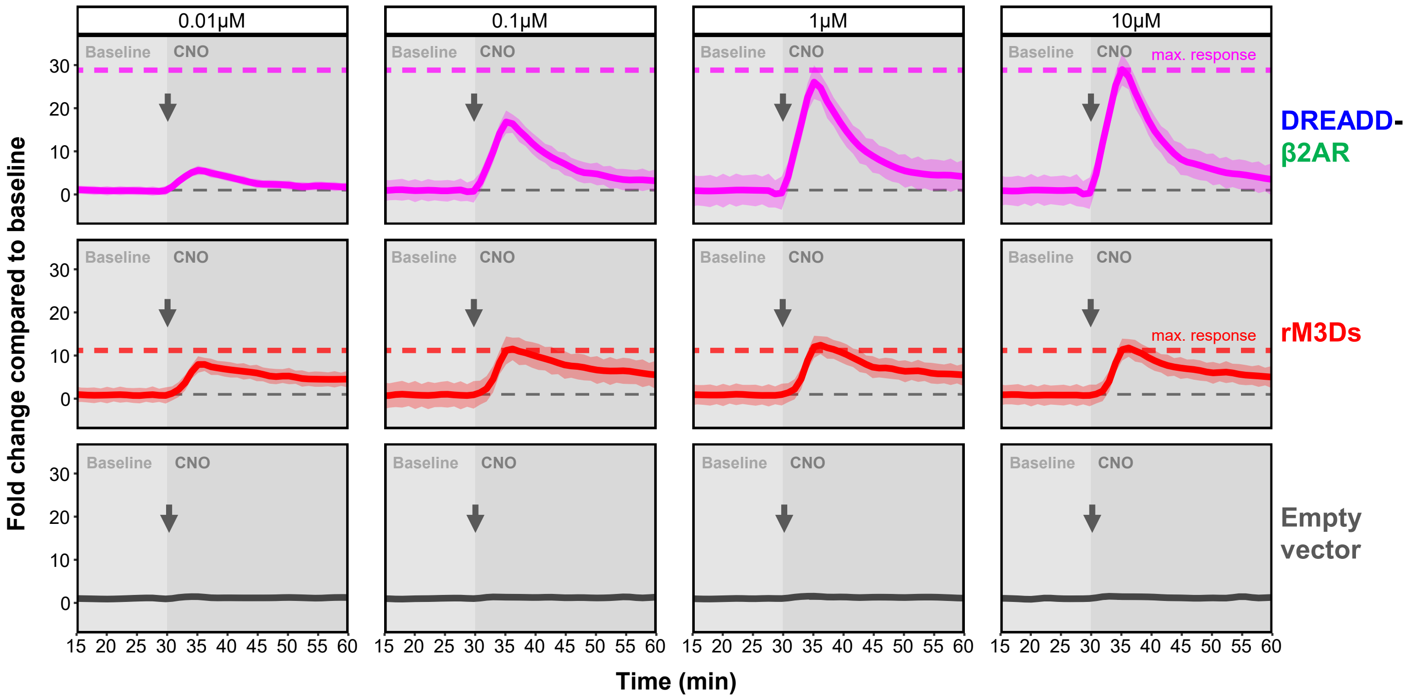

c *Endogenous β2AR contributes only partially to the response of β2AR-transfected HEK cells*

Dose-dependent contribution to SRE reporter (MAPK) inhibition evaluated with two β2AR ligands

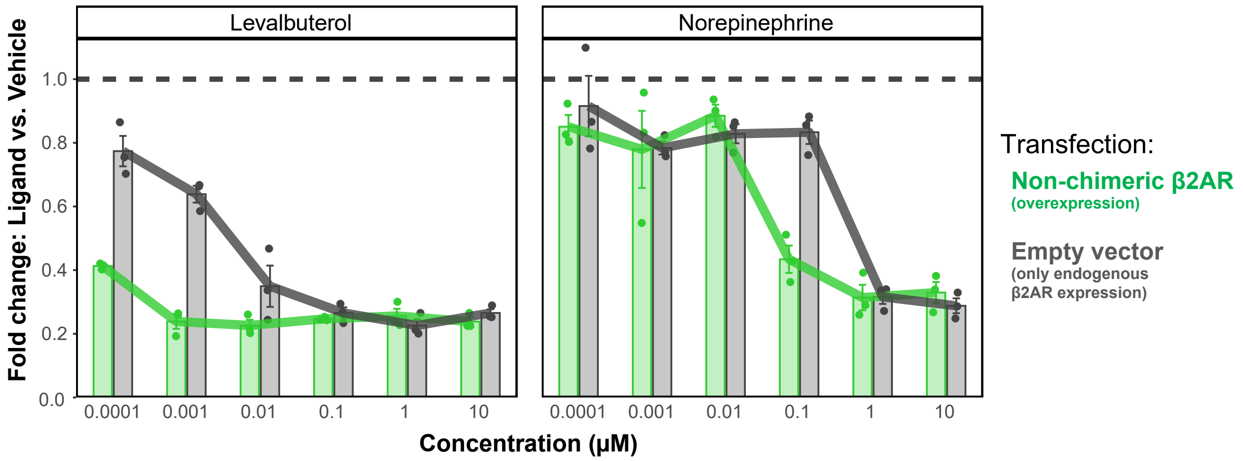

Supplementary Figure S3: Endogenous β2AR contributes only partially in β2AR-transfected HEK cells and the CNO response of DREADD-β2AR and rM3Ds is saturated at 10µM.

a: Real-time measurement of cAMP-dependent luciferase activity in HEK cells transfected with non-chimeric β2AR (green) or empty vector (grey). Baseline measurements of 30 minutes (first 15 minutes not shown) followed by application of β2AR agonists levalbuterol or norepinephrine (grey arrow for onset). Measure of center: Mean fold change compared to baseline mean (dashed line) in the same experimental repetition. Ribbons: 95% confidence intervals. N = three experimental repetitions. Source data are provided as a Source Data file. **b:** Real-time measurement of cAMP-dependent luciferase activity in HEK cells transfected with DREADD-β2AR (magenta), rM3Ds (red), or empty vector (grey). Baseline measurements of 30 minutes (first 15 minutes not shown) followed by CNO application (grey arrow for onset) at concentrations ranging from 0.01µM to 10µM. Measure of center: Mean fold change compared to baseline mean (dashed line) in the same experimental repetition. Maximum responses are indicated by magenta (DREADD-β2AR) and red (rM3Ds) dashed lines. Ribbons: 95% confidence intervals. N = four (DREADD-β2AR: all concentrations), three (rM3Ds, all concentrations), six (Empty vector: 0.01-1µM), or seven (Empty vector: 10µM) experimental repetitions. Source data are provided as a Source Data file. **c:** Graph shows endpoint measurement of SRE-dependent luciferase activity in HEK cells transfected with non-chimeric β2AR (green) or empty vector (grey). Dashed line: level of vehicle control. Error bars: standard error of the mean. N = three experimental repetitions. Source data are provided as a Source Data file.

Supplementary Figure S4

Images of Western blot membranes

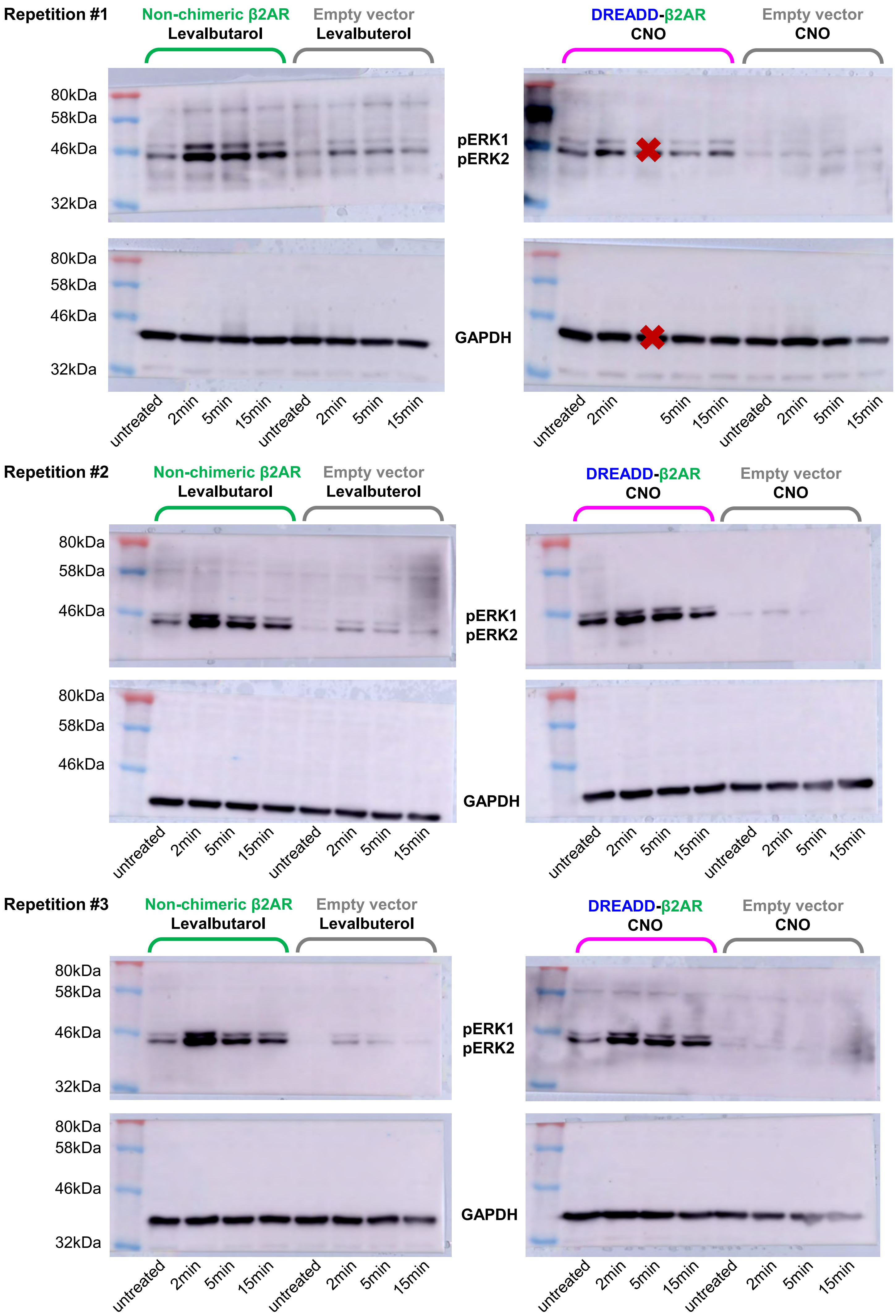

Supplementary Figure S4: Western blot analysis of ERK1/2 phosphorylation.

Summary of all Western blots for phosphorylation analysis of extracellular signal-regulated kinases 1 and 2 (ERK1/2) in untreated, levalbuterol- or CNO-treated HEK cells transfected with non-chimeric β2AR (green), DREADD-β2AR (magenta), or empty vector (grey). Densitometry analysis shown in Figure 4c. Red cross: lanes excluded due to incorrect sample loading.

Supplementary Figure S5

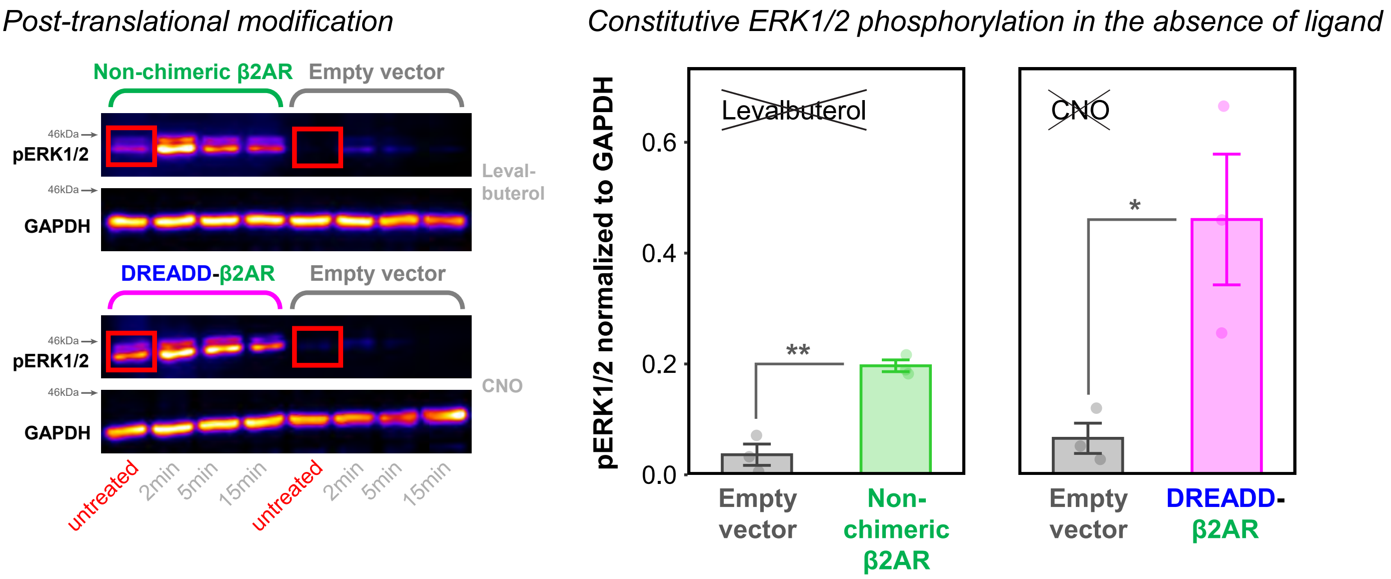

Supplementary Figure S5: Constitutive activity drives ERK1/2 phosphorylation in the absence of ligand stimulation.

Comparison of phosphorylated extracellular signal-regulated kinases 1 and 2 (ERK1/2) in untreated HEK cells transfected with non-chimeric β2AR, DREADD-β2AR, or empty vector. Left: Western blot for pERK1/2 and GAPDH (loading control). The anti-pERK1/2 antibody results in an upper band for pERK1 (44kDa) and a lower band for pERK2 (42kDA). Red rectangles indicate the bands in untreated samples. Right: Densitometry analysis of combined pERK1/2 normalized to GAPDH. Error bars: standard error of the mean. Two-sided two-sample T-test: $p^{**} < 0.01$; $p^{*} < 0.05$. Exact p-values of individual T-tests without multiple testing correction: $p = 0.002$ (Non-chimeric β2AR vs. Empty vector); $p = 0.03$ (DREADD-β2AR vs. Empty vector). N = three experimental repetitions. Source data are provided as a Source Data file.

Supplementary Figure S6

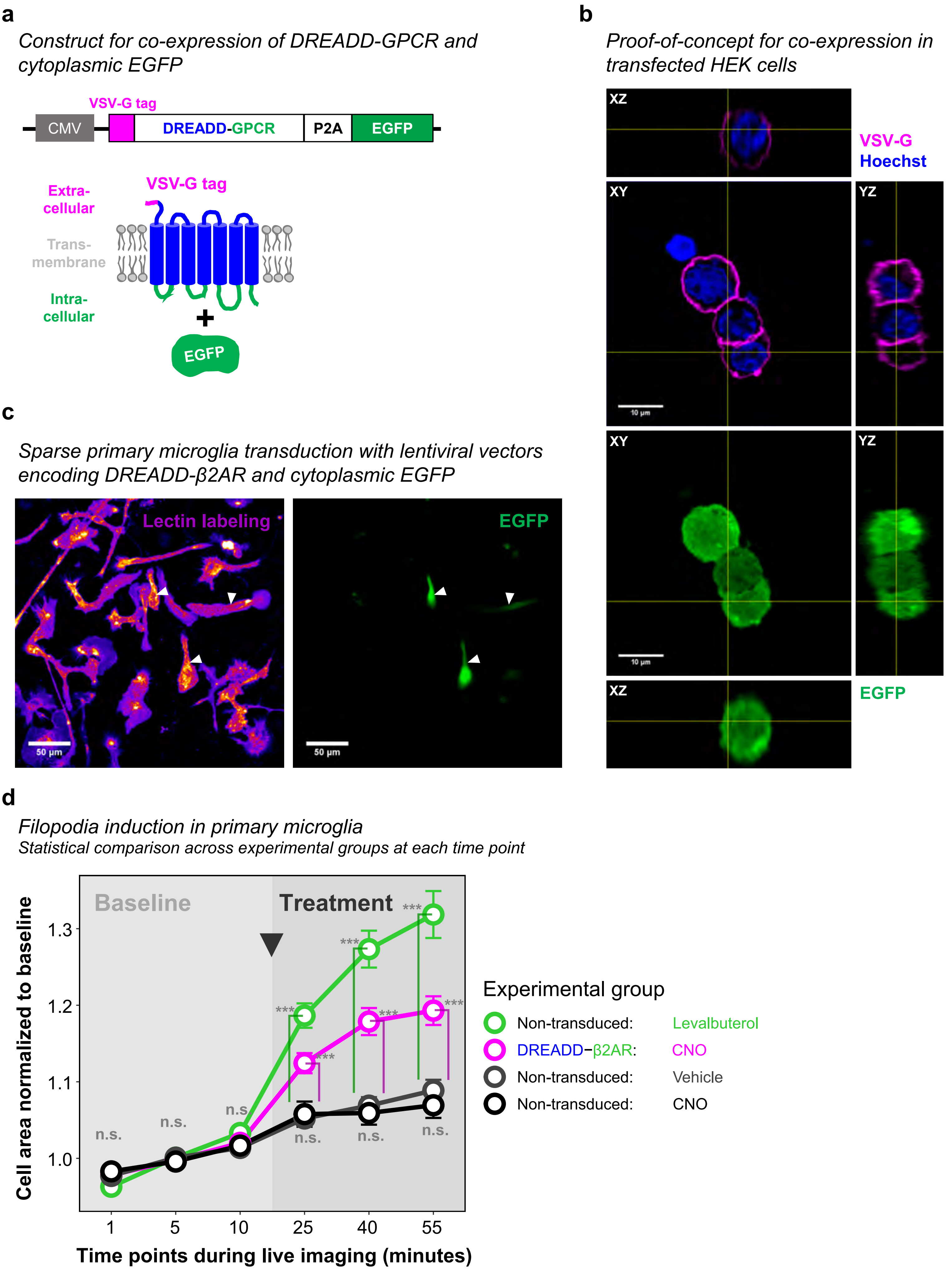

Supplementary Figure S6: Vector validation for bicistronic expression of DREADD-GPCRs and EGFP.

a: Schematic of a bicistronic vector with human cytomegalovirus (CMV) promoter, VSV-G epitope, DREADD-GPCR, the self-cleaving P2A peptide sequence, and EGFP. Transfected cells express the GPCR on the cell surface and EGFP in the cytoplasm. **b:** Orthogonal view of HEK cells transfected with DREADD-β2AR-P2A-EGFP immunostained for the VSV-G tag under non-permeabilizing conditions. Magenta: VSV-G tag. Green: EGFP. Blue: nuclear staining with Hoechst. **c:** Unstained primary microglia seven days after transduction with a lentiviral vector encoding DREADD-β2AR-P2A-EGFP at a multiplicity of infection (MOI) of 3. Left: labeling with fluorophore-conjugated tomato lectin, displayed with intensity-based color code (purple-red-yellow). Right: EGFP. **d:** Analysis of filopodia formation in primary microglia using lentiviral expression of DREADD-β2AR-P2A-EGFP. Graph combines all panels from Figure 6e and shows statistical comparison between experimental groups within each time point. Error bars: standard error of the mean. Linear regression analysis with two-sided post-hoc comparisons corrected for multiple testing: $p^{***} < 0.001$; $p^{n.s.} > 0.05$. Exact p-values for selected comparisons at the 25min time point: p = 1,00 (Non-transduced: Vehicle vs. Non-transduced: CNO); p < 0.001 (Non-transduced: Vehicle vs. Non-transduced: Levalbuterol); p < 0.001 (Non-transduced: Vehicle vs. DREADD-β2AR:CNO). Selected comparisons at the 40min time point: p = 0.99 (Non-transduced: Vehicle vs. Non-transduced: CNO); p < 0.001 (Non-transduced: Vehicle vs. Non-transduced: Levalbuterol); p < 0.001 (Non-transduced: Vehicle vs. DREADD-β2AR: CNO). Selected comparisons at the 55min time point: p = 0.99 (Non-transduced: Vehicle vs. Non-transduced: CNO); p < 0.001 (Non-transduced: Vehicle vs. Non-transduced: Levalbuterol); p < 0.001 (Non-transduced: Vehicle vs. DREADD-β2AR: CNO). See Supplementary Data 3 for exact p-values of all comparisons. N = 30 (Non-transduced: Levalbuterol), 32 (DREADD-β2AR: CNO), 50 (Non-transduced: Vehicle), 30 (Non-transduced: CNO) cells examined over three, ten, nine, and eight experiments, respectively. Source data are provided as a Source Data file.

Supplementary Figure S7

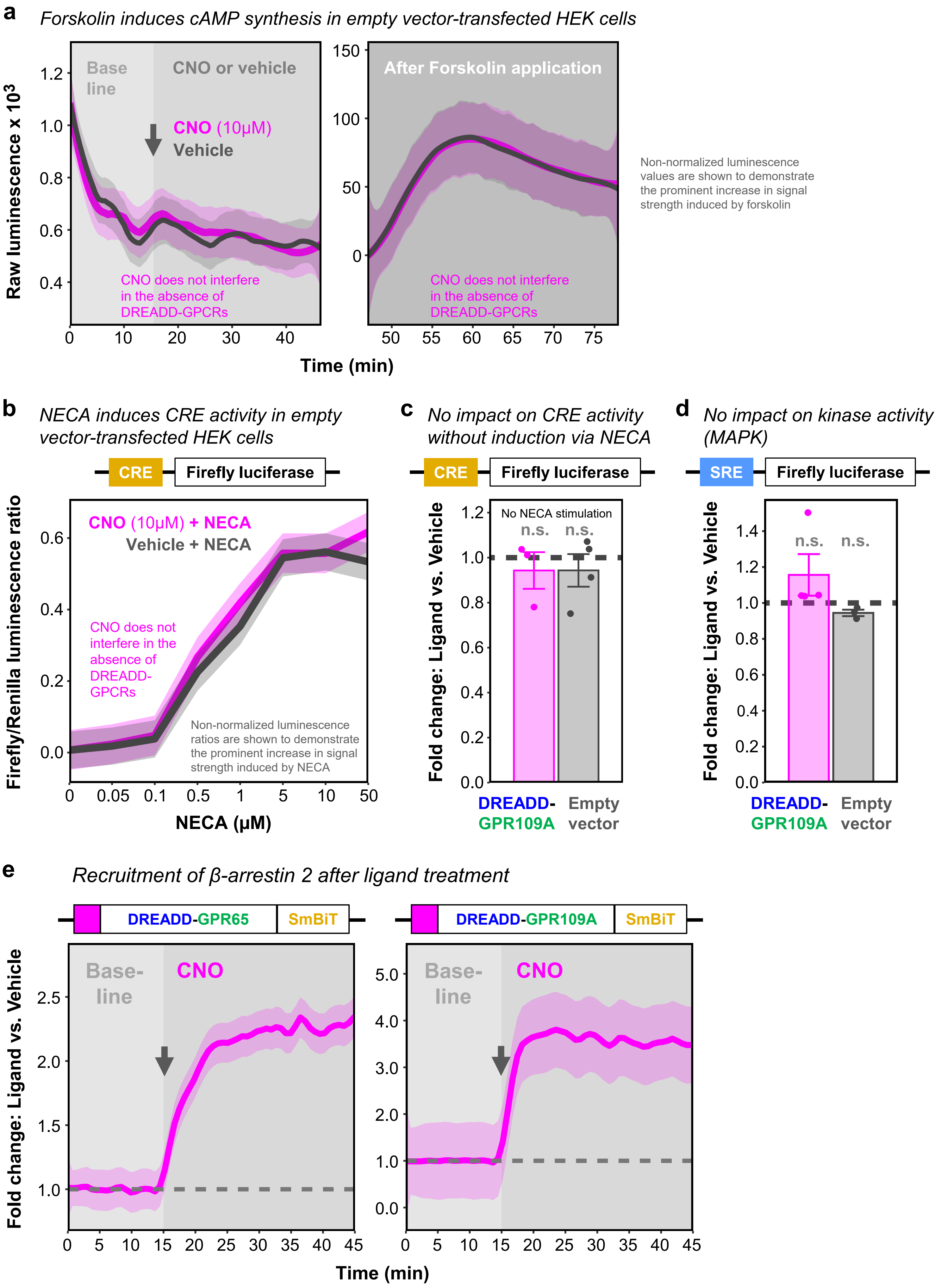

Supplementary Figure S7: Additional controls and validations.

a: CNO does not impair forskolin-induced cAMP synthesis in the absence of DREADD-GPCRs. Real-time measurement of cAMP-dependent luciferase activity in HEK cells transfected with empty vector. Baseline measurements followed by application of CNO or vehicle (grey arrow for onset) and forskolin (second panel). CNO treatment is shown in magenta and vehicle in grey. Measure of center: Mean raw luminescence units. Non-normalized raw values are used for this visualization to exemplify the strong signal increase upon forskolin application. Ribbons: 95% confidence intervals. N = five experimental repetitions. Source data are provided as a Source Data file. **b:** CNO does not impact NECA-induced transcription from a cAMP responsive element (CRE) in the absence of DREADD-GPCRs. Endpoint measurement of cAMP responsive element (CRE)-dependent firefly luciferase activity in HEK cells transfected with empty vector. Treatment with increasing concentrations of NECA in presence of either CNO (magenta) or vehicle (grey). Graph shows non-normalized firefly/renilla luciferase luminescence ratios to exemplify the signal increase upon NECA application. Ribbons: standard error of the mean. N = three technical replicates from one experiment. Source data are provided as a Source Data file. **c:** DREADD-GPR109A does not detectably inhibit CRE reporter activity upon CNO application without inducing CRE through NECA. Endpoint measurement of CRE-dependent luciferase activity in HEK cells transfected with DREADD-GPR109A (magenta) or empty vector (grey). Ligand stimulation with CNO. Dashed line: level of the respective vehicle control. Error bars: standard error of the mean. One-sample T-test: $p^{n.s.} > 0.05$. N = three (DREADD-GPR109A) or four (Empty vector) experimental repetitions. Source data are provided as a Source Data file. **d:** DREADD-GPR109A does not influence SRE reporter activity upon CNO application. Endpoint measurement of serum responsive element (SRE)-dependent luciferase activity in HEK cells transfected with DREADD-GPR109A (magenta) or empty vector (grey). Ligand stimulation with CNO. Dashed line: level of the respective vehicle control. Error bars: standard error of the mean. One-sample T-test: $p^{n.s.} > 0.05$. N = four (DREADD-GPR109A) or three (Empty vector) experimental repetitions. Source data are provided as a Source Data file. **e:** DREADD-GPR65 and GPR109A recruit β-arrestin 2. Real-time measurement of β-arrestin 2 recruitment in HEK cells transfected with DREADD-GPR65 (left) or DREADD-GPR109A (right). Baseline measurements followed by CNO application (grey arrow shows onset). Measure of center: Mean of baseline-normalized fold change compared to vehicle (dashed line) in the same experimental repetition. Ribbons: 95% confidence intervals. N = three experimental repetitions. Source data are provided as a Source Data file.

Supplementary Figure S8

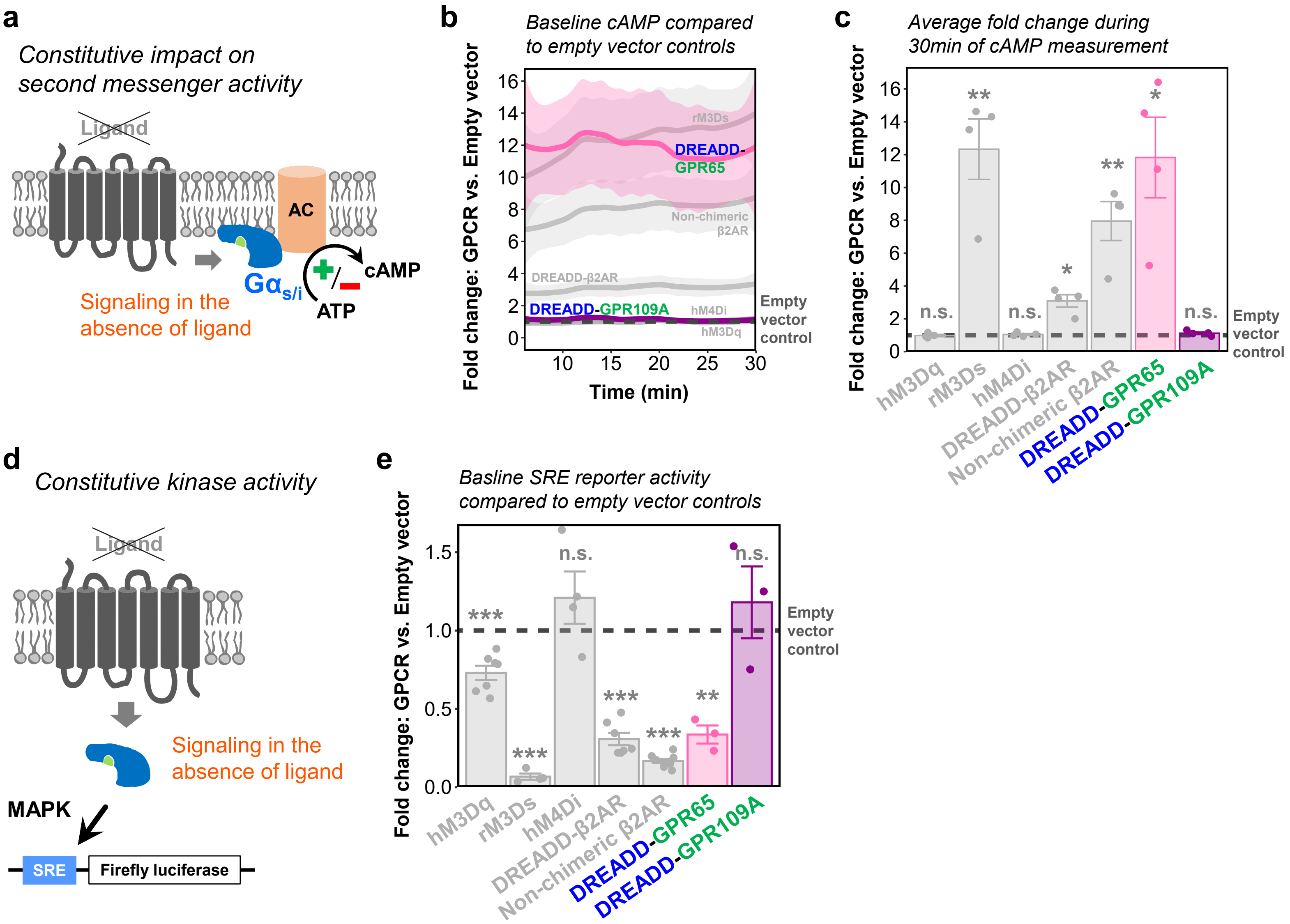

Supplementary Figure S8: Evaluating constitutive activity of DREADD-GPR65 and DREADD-GPR109A.

a: Schematic of GPCR impacting baseline cAMP levels through constitutive activity. **b:** Real-time measurement of cAMP-dependent luciferase activity during a 30min baseline in HEK cells transfected with DREADD-GPR65 (pink) and DREADD-GPR109A (purple). Previously evaluated GPCRs (light grey) from Figure 3 are included for comparison. Measure of center: Mean fold change compared to empty vector (dashed line) in the same experimental repetition. Ribbons: 95% confidence intervals. N = four experimental repetitons. Source data are provided as a Source Data file. **c:** Graph shows average fold changes compared to empty vector control during the 30min measurement in **b**. Dashed line: level of empty vector control. Error bars: standard error of the mean. Two-sided one-sample T-test for comparing to a mean of 1 representing the empty vector control: $p^{***} < 0.001$; $p^{**} < 0.01$; $p^{n.s.} > 0.05$. Exact p-values of individual T-tests without multiple testing correction: p = 0.81 (hM3Dq); p = 0.009 (rM3Ds); p = 0.48 (hM4Di); p = 0.01 (DREADD-β2AR); p = 0.009 (Non-chimeric β2AR); p = 0.02 (DREADD-GPR65); p = 0.20 (DREADD-GPR109A). N = four experimental repetitions. Source data are provided as a Source Data file. **d:** Schematic of GPCR with constitutive activity impacting baseline MAPK signaling measured through an SRE reporter. **e:** Endpoint measurement of SRE-dependent luciferase activity in transfected HEK cells. Dashed line: level of empty vector control. Error bars: standard error of the mean. Two-sided one-sample T-test for comparing to a mean of 1 representing the empty vector control: $p^{***} < 0.001$; $p^{**} < 0.01$; $p^{n.s.} > 0.05$. Exact p-values of individual T-tests without multiple testing correction: p < 0.001 (hM3Dq); p < 0.001 (rM3Ds); p = 0.30 (hM4Di); p < 0.001 (DREADD-β2AR); p < 0.001 (Non-chimeric β2AR); p = 0.007 (DREADD-GPR65); p = 0.52 (DREADD-GPR109A). N = seven (hM3Dq, DREADD-β2AR), four (rM3Ds, hM4Di), nine (Non-chimeric β2AR), or three (DREADD-GPR65, DREADD-GPR109A) experimental repetitions. Source data are provided as a Source Data file.

Supplementary Figure S9

Confirming DREADD-GPCR surface expression in HMC3 cells

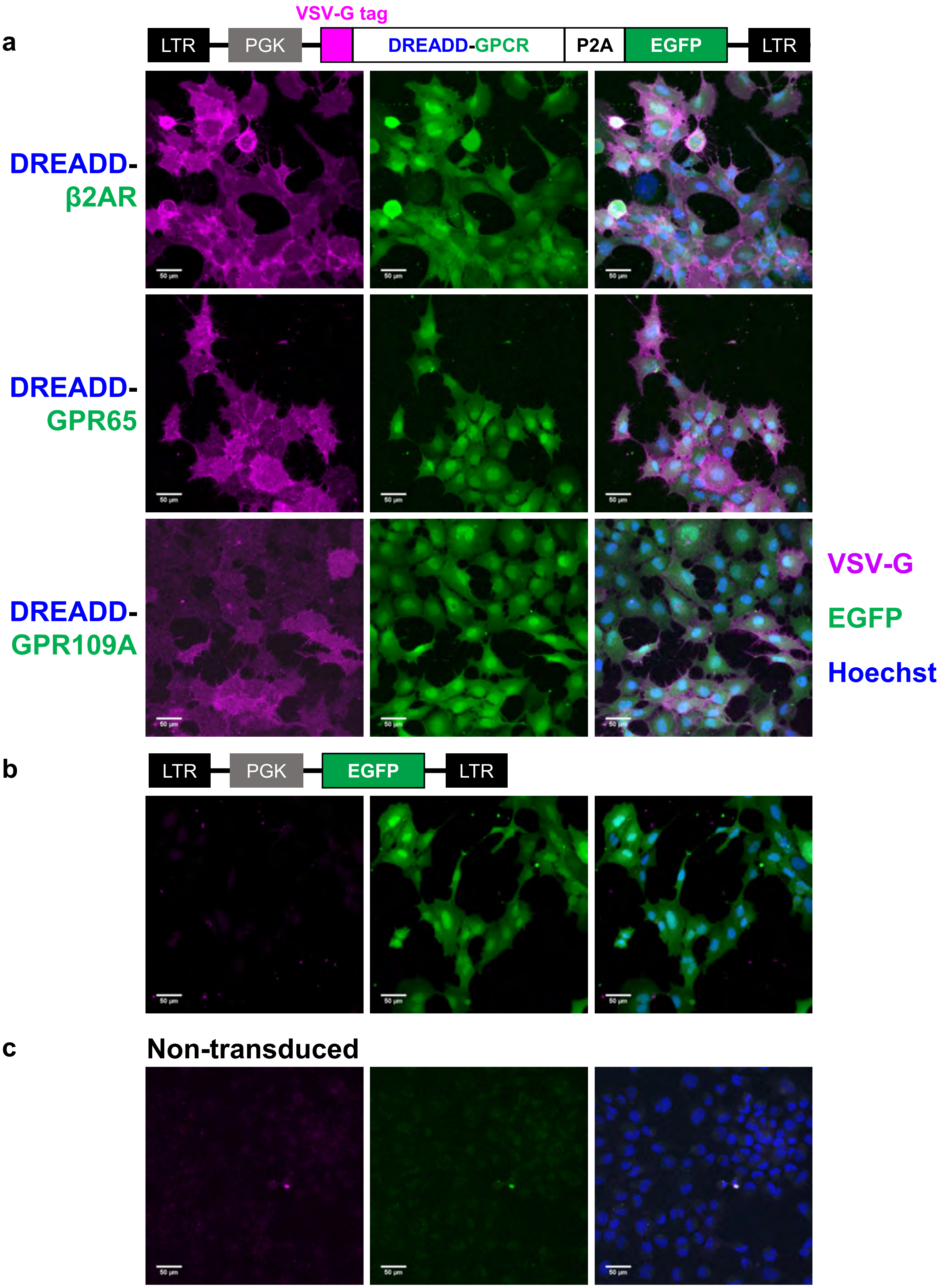

Supplementary Figure S9: Stable expression of DREADD-based chimeras in HMC3 cells through lentiviral vectors.

a: Schematic of lentiviral vector with DREADD-GPCR-P2A-EGFP flanked by long terminal repeats (LTRs) and expression driven by the phosphoglycerate kinase (PGK) promoter. Images show maximum intensity projections of HMC3 cells immunostained for the VSV-G tag under non-permeabilizing conditions. Cells were either transduced with DREADD-β2AR, DREADD-GPR65, or DREADD-GPR109A (**a**); a control vector encoding only EGFP (**b**); or remained non-transduced (**c**). Magenta: VSV-G. Green: EGFP. Blue: nuclear staining with Hoechst.

Supplementary Figure S10

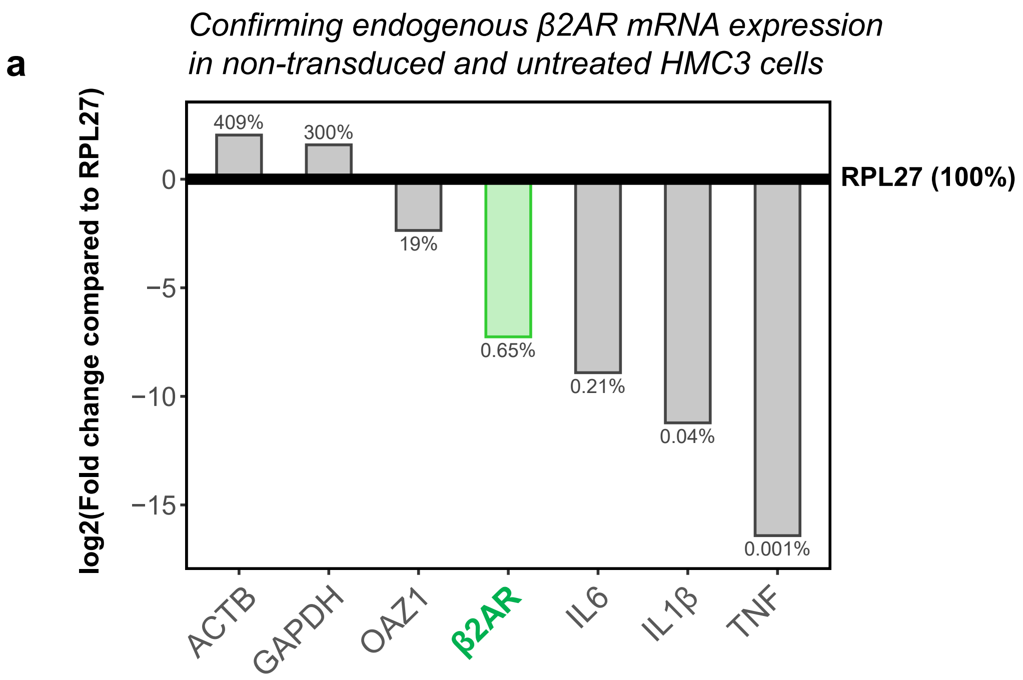

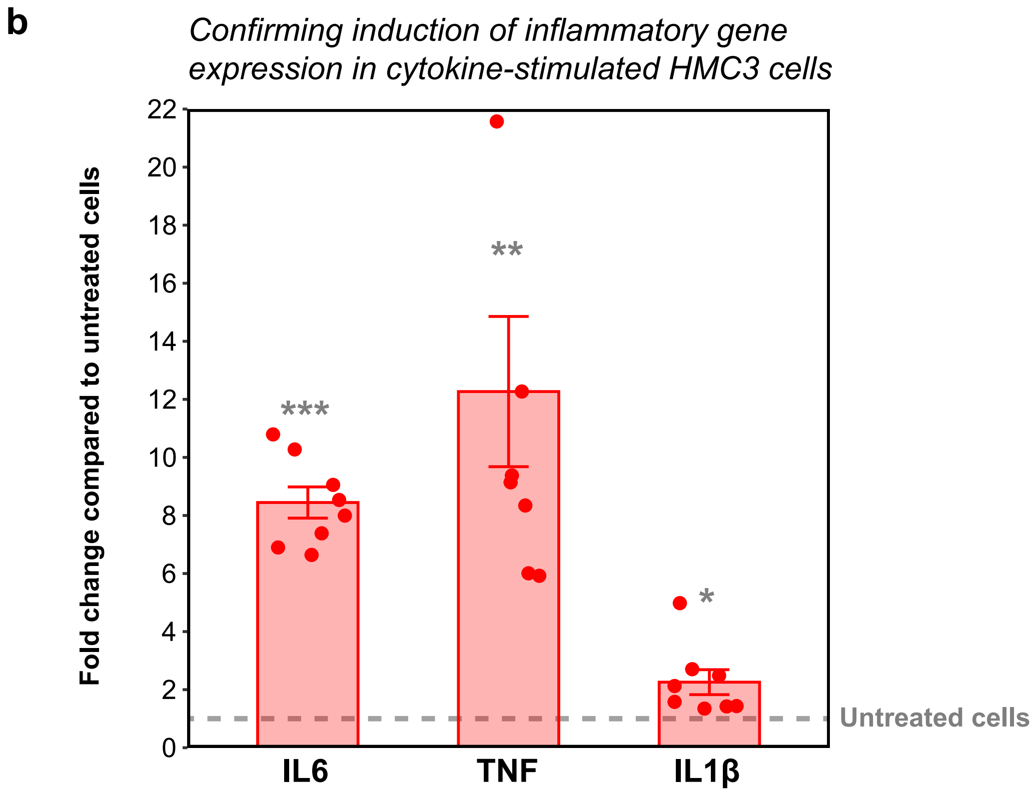

Supplementary Figure S10: HMC3 cells endogenously express β2AR and respond to the recombinant cytokines IFNγ and IL1β.

a: Quantitative reverse transcription PCR (RT-qPCR)-based comparison of gene expression in non-transduced and untreated HMC3 cells. Bars: log2 fold changes compared to RPL27 and are based on the mean of three technical replicates from one experiment. Relative transcript abundance is shown as a percentage of the reference gene RPL27 (100%). ACTB, β-actin. GAPDH, glyceraldehyde-3-phosphate dehydrogenase. OAZ1, ornithine decarboxylase antizyme 1. RPL27, ribosomal protein L27. Source data are provided as a Source Data file. **b:** RT-qPCR of non-transduced HMC3 cells exposed to recombinant interferon γ (IFNγ) and interleukin 1β (IL1β). Expression values of interleukin 6 (*IL6*), tumor necrosis factor (*TNF*), and *IL1β* shown as fold change compared to untreated cells (dashed line). Error bars: standard error of the mean. Two-sided one-sample T-test comparing to a mean of 1 representing untreated cells: $p^{***} < 0.001$; $p^{*} < 0.01$; $p^{*} < 0.05$. Exact p-values of individual T-tests without multiple testing correction: $p < 0.001$ (*IL6*); $p = 0.003$ (*TNF*); $p = 0.02$ (*IL1β*). N = eight experimental repetitions. Source data are provided as a Source Data file.

Supplementary Figure S11

a *HMC3 cells display spontaneous Ca^{2+} events during 6 minutes of Ca^{2+} imaging*

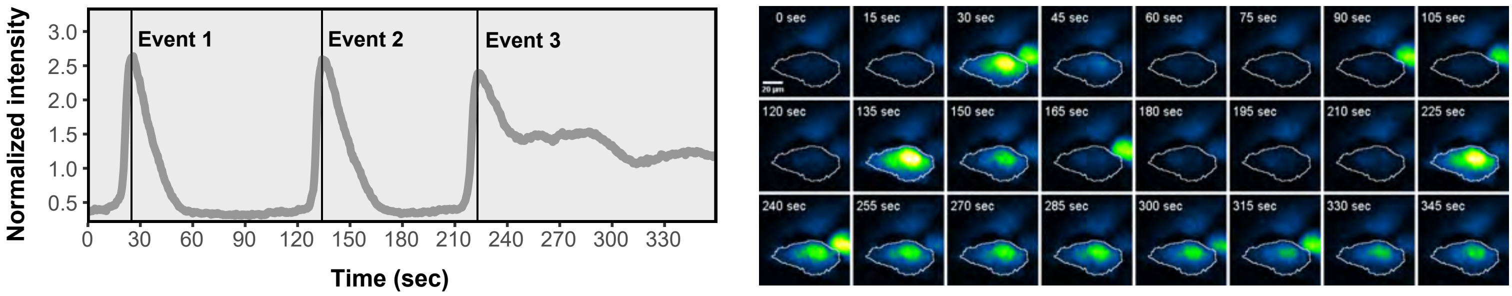

b *Fluorescence intensity of 32 cells per panel during 6 minutes of imaging (drug stimulation after 180 seconds)*

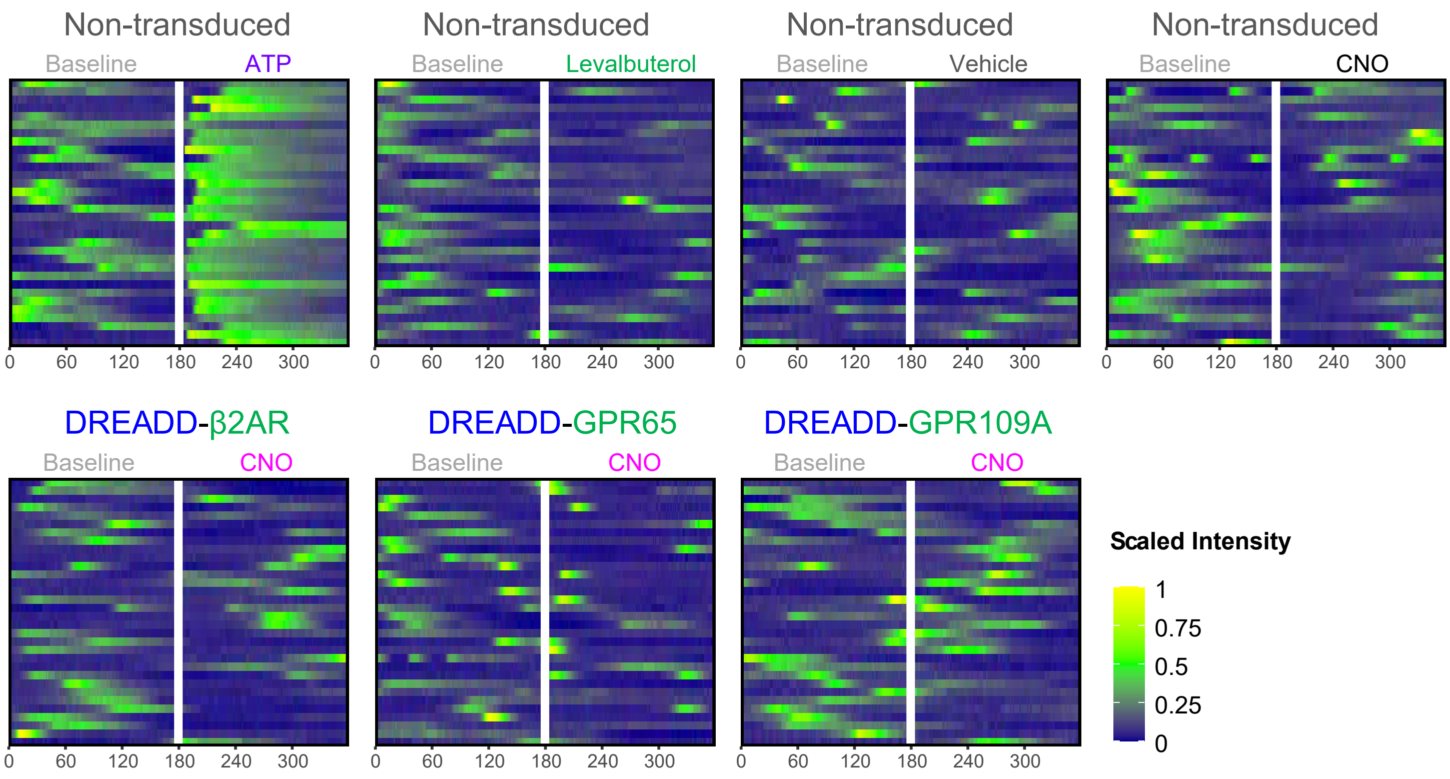

c *Sum of calcium events confirms response to ATP but not to endogenous β2AR or DREADD-GPCR stimulation*

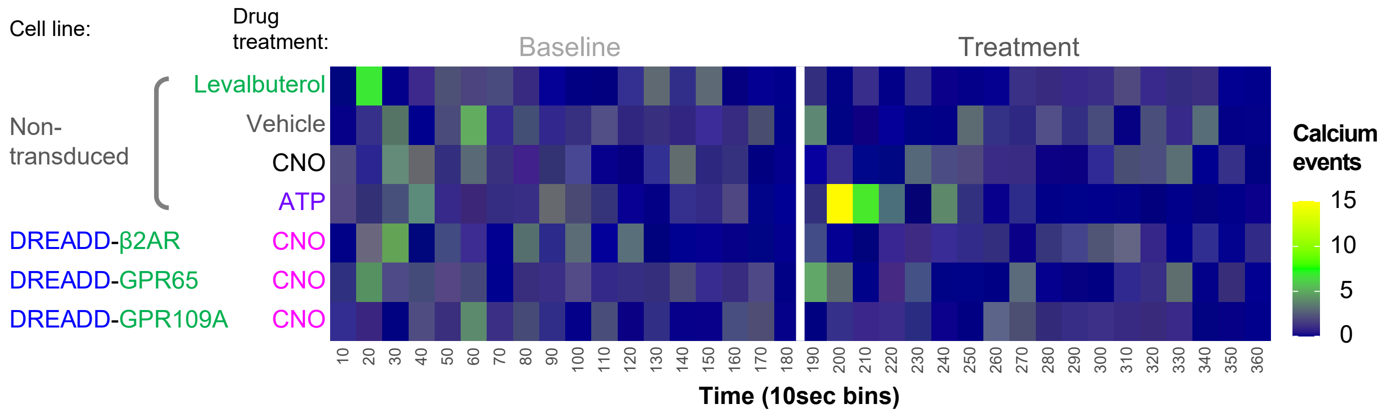

Supplementary Figure S11: β2AR and DREADD-GPCRs do not induce Ca^{2+} signaling in HMC3 cells.

a: Example of HMC3 cells displaying spontaneous Ca^{2+} events during six minutes of Ca^{2+} imaging. Left: graph shows Ca^{2+}-dependent fluorescence intensity normalized to the mean intensity of the cell throughout the recording. Ca^{2+} events are software-detected. Source data are provided as a Source Data file. Right: consecutive images of the analyzed cell at representative time points. Ca^{2+}-dependent fluorescence is displayed through an intensity-based color code (blue-green-yellow). **b:** Each panel shows an HMC3 cell line treated with either ATP (positive control), levalbuterol, vehicle, or CNO. Each row in a panel is an individual cell. Ca^{2+}-dependent fluorescence intensity is scaled per panel. White vertical line indicates drug application time point after three minutes baseline. Source data are provided as a Source Data file. **c:** Graph shows sum of software-detected Ca^{2+} events from all cells per condition (panels in **b**) across time in 10 second-bins. White vertical line indicates drug application time point. Source data are provided as a Source Data file.

Supplementary Figure S12

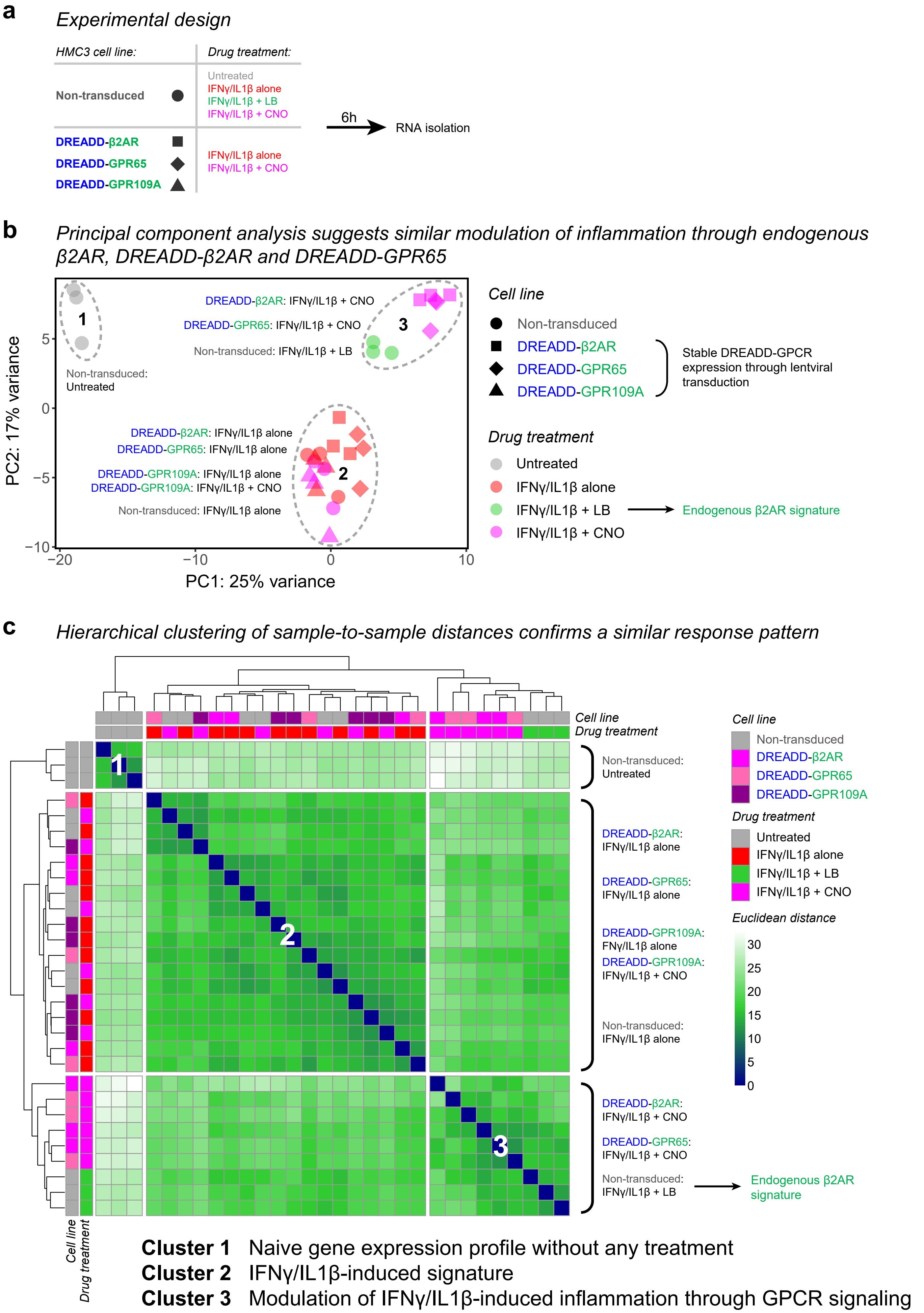

Cluster 1 Naive gene expression profile without any treatment
Cluster 2 IFNγ/IL1β-induced signature
Cluster 3 Modulation of IFNγ/IL1β-induced inflammation through GPCR signaling

Supplementary Figure S12: Principal component analysis and hierarchical clustering reveal similar response patterns.

a: Experimental layout for next generation mRNA sequencing of HMC3 cell lines. IFNγ: interferon γ. IL1β: interleukin 1β. LB: levalbuterol. **b:** Principal component analysis including all genes. Source data are provided as a Source Data file. **c:** Hierarchical clustering of Euclidean sample-to-sample distances based on all genes. Cluster numbers correspond to clusters in the principal component analysis (**a**). Source data are provided as a Source Data file.

Supplementary Figure S13

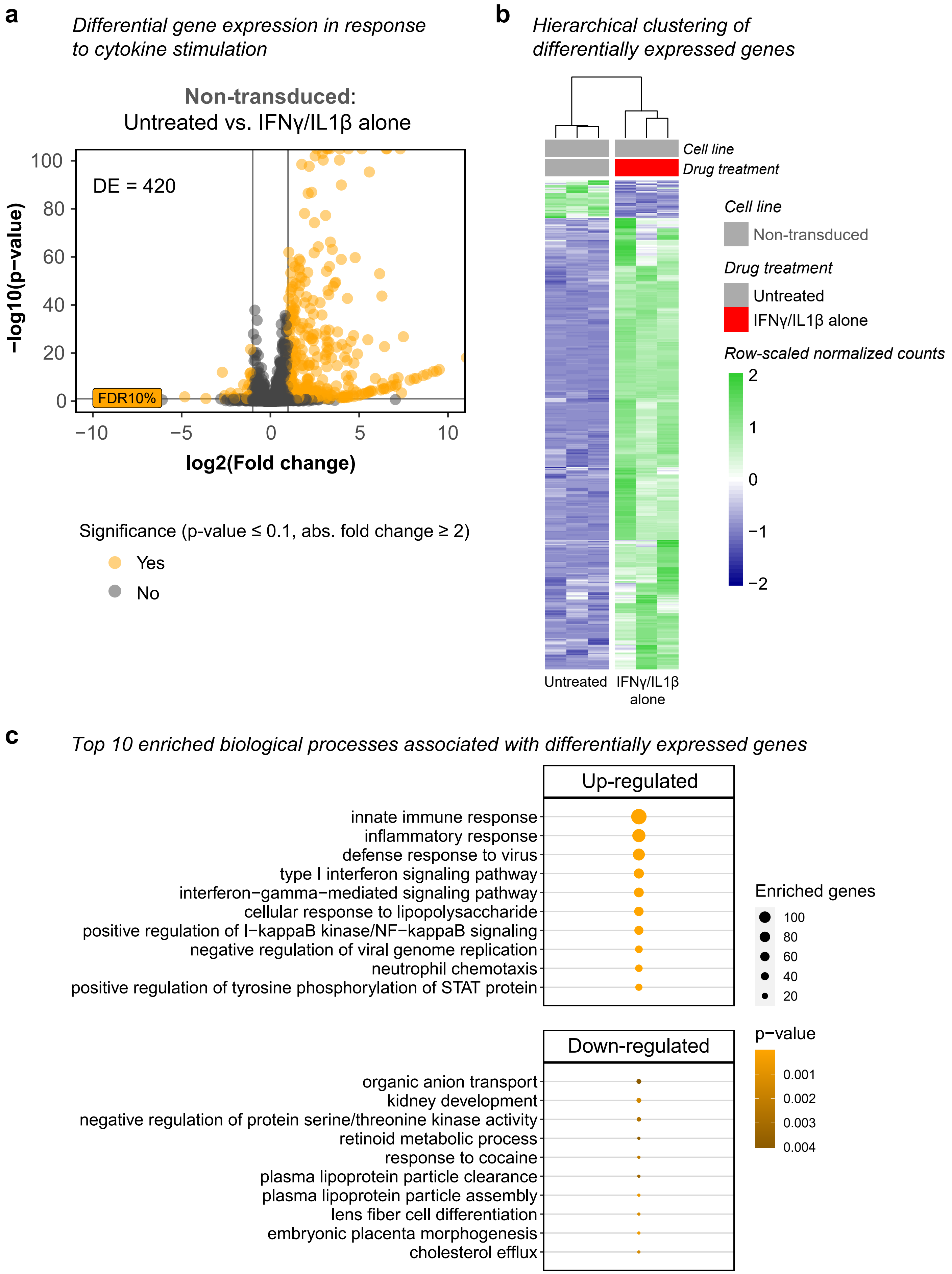

Supplementary Figure S13: Confirmation of successfully induced inflammation in HMC3 cells treated with the recombinant cytokines IFNγ and IL1β.

a: Vulcano plot of next generation mRNA sequencing data comparing non-transduced and untreated HMC3 cells to stimulation with IFNγ/IL1β alone. Graph shows individual genes (points), their log2 fold change and p-value. DE: number of differentially expressed genes (orange data points) defined by $p < 0.1$ and absolute linear fold change > 2. Horizontal lines: false discovery rate (FDR) cutoff of 10% ($p < 0.1$). Vertical lines: linear fold change cutoff for downregulation (< -2) and upregulation (> 2), respectively. Source data are provided as a Source Data file. **b:** Hierarchical clustering of samples (columns) across all differentially expressed genes (rows) based on row-scaled normalized counts. Source data are provided as a Source Data file. **c:** Gene ontology enrichment analysis showing the top 10 biological processes associated with differentially expressed up- and downregulated genes. Dot size: number of enriched genes associated with a biological process. Dot color: p-value of enrichment analysis. Source data are provided as a Source Data file.

Supplementary Figure S14

Top 6 enriched biological processes associated with differentially expressed gene clusters

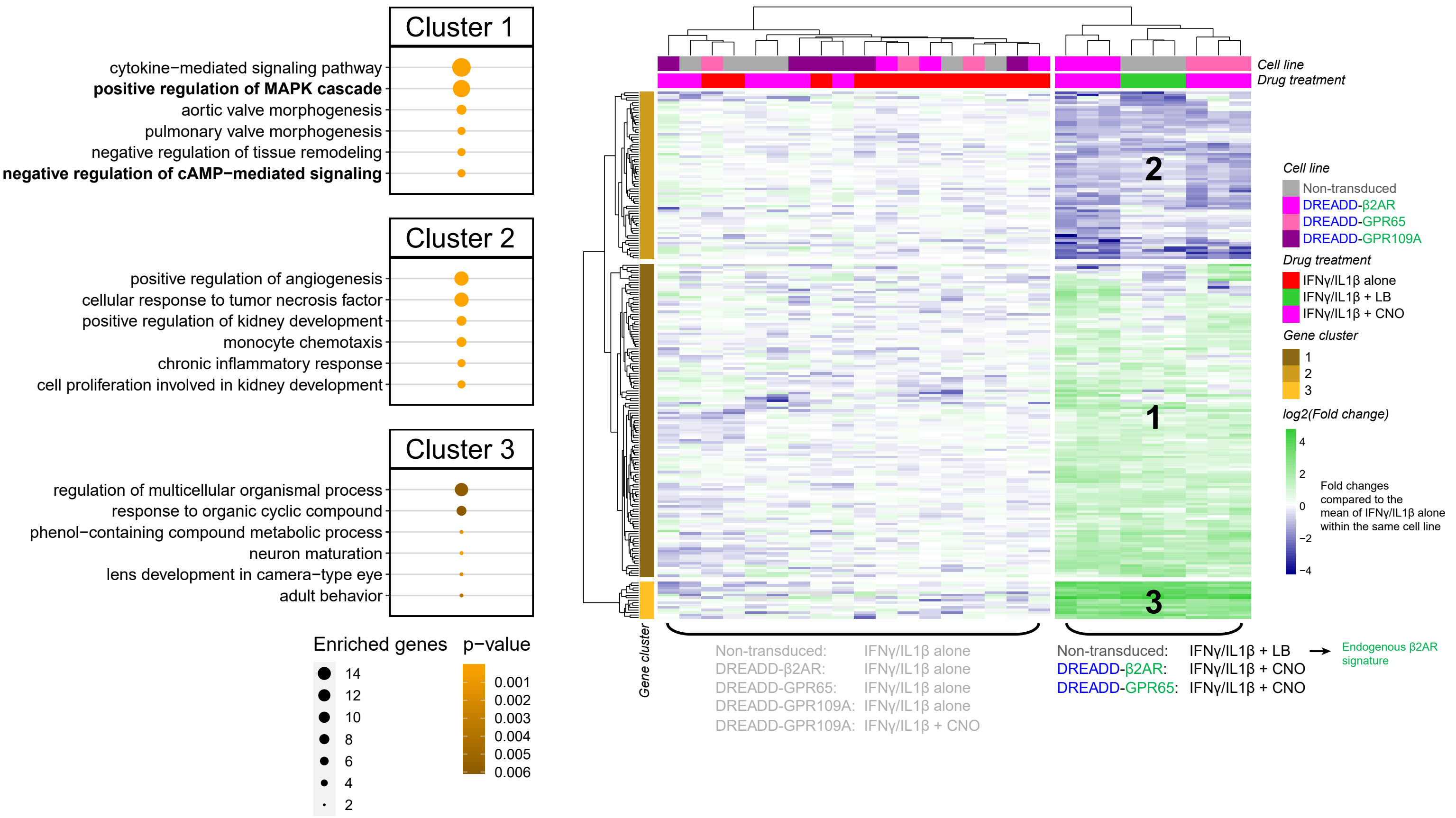

Supplementary Figure S14: Gene ontology enrichment analysis of gene clusters. Identification of three gene clusters through hierarchical clustering of HMC3 samples (columns) across all differentially expressed genes (rows) in Figure 9a-c based on their log2 fold changes. Upregulation (green) and downregulation (blue) compared to IFNγ/IL1β alone in the same cell line. Dot size: number of enriched genes associated with a biological process. Dot color: p-value of enrichment analysis (Fisher test). Source data are provided as a Source Data file.

Supplementary Figure S15

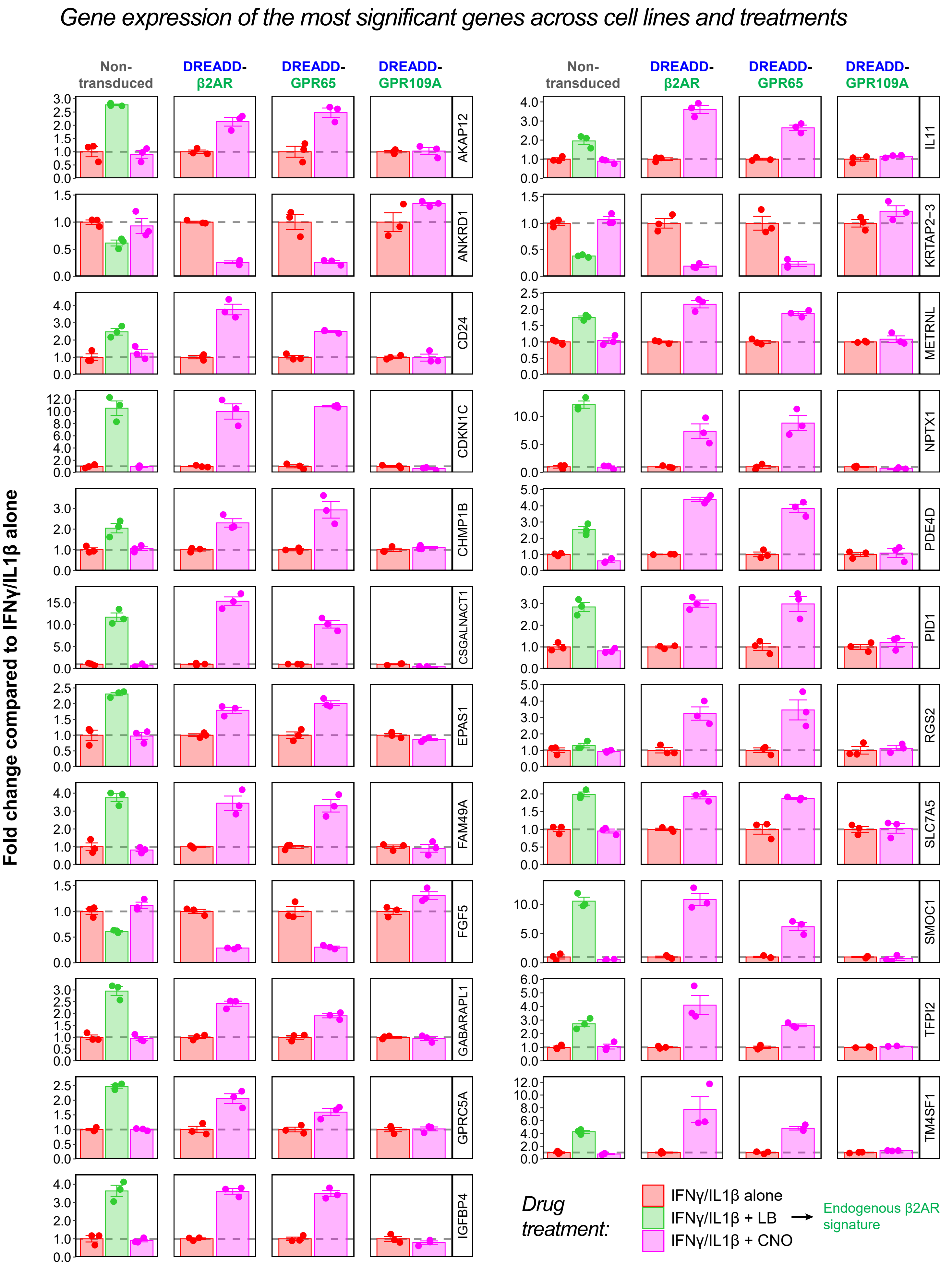

Supplementary Figure S15: Bar graphs for comparison of the top differentially expressed genes.

Graphs show linear fold changes compared to IFNγ/IL1β alone within each HMC3 cell line. Genes are selected based on smallest adjusted p-values from comparisons in Figure 9a-c. Dashed line: level of IFNγ/IL1β alone. Error bars: standard error of the mean. N = three experimental repetitions. Source data are provided as a Source Data file.

Supplementary Figure S16

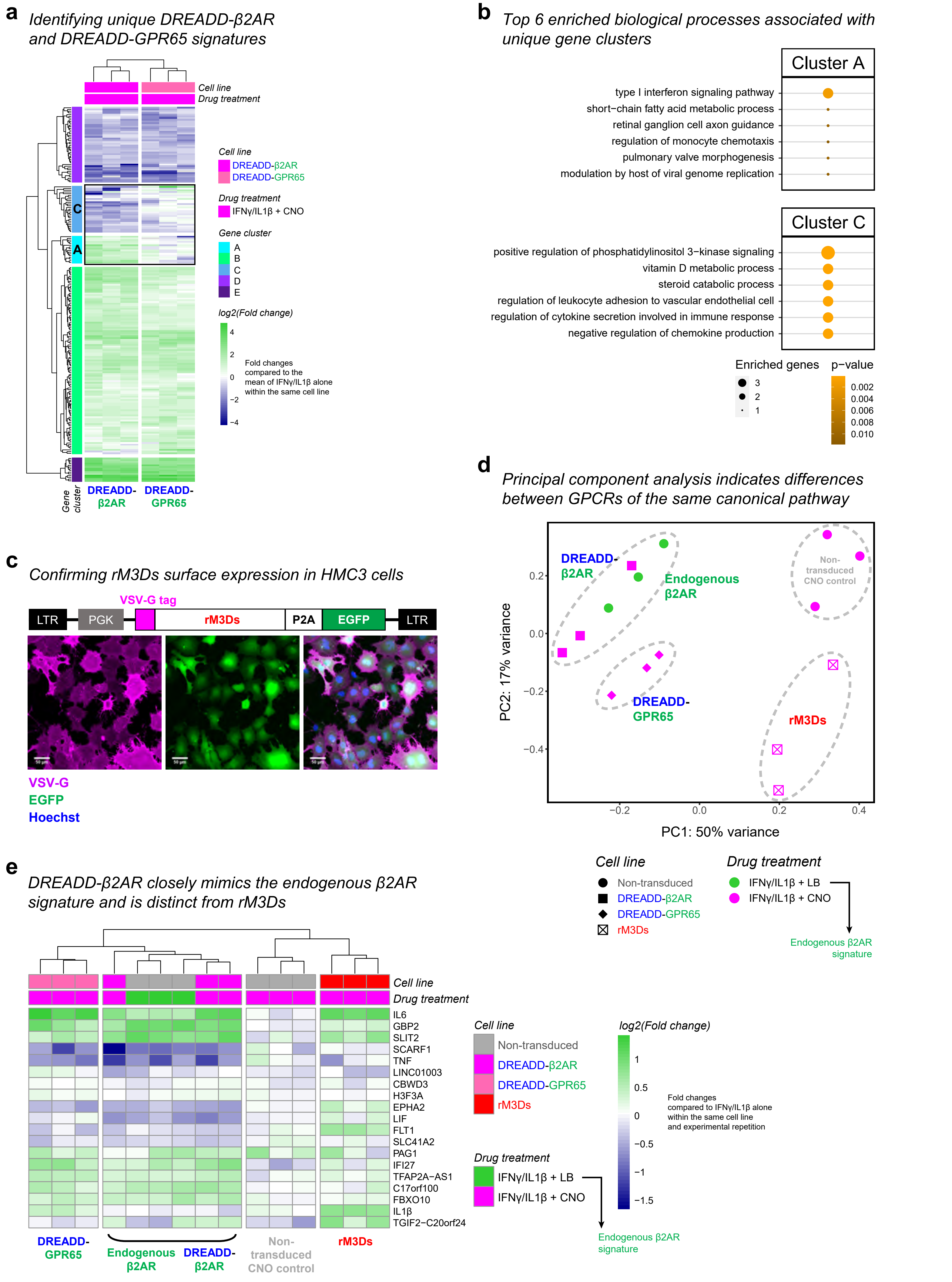

Supplementary Figure S16: GPCRs coupled to the same canonical pathway are capable of inducing subtle but distinct transcriptional patterns.

a: mRNA sequencing-based identification of five gene clusters through hierarchical clustering of HMC3 cell samples (columns) across all differentially expressed genes (rows) in Figure 9a-c based on their log2 fold changes. Upregulation (green) and downregulation (blue) compared to IFNγ/IL1β alone in the same cell line. Black rectangle highlights gene clusters A and C suggestive of a unique pattern between DREADD-β2AR and DREADD-GPR65. Source data are provided as a Source Data file. **b:** Gene ontology enrichment analysis of the top 6 biological processes associated with genes in cluster A and C. Dot size: number of enriched genes associated with a biological process. Dot color: p-value of enrichment analysis (Fisher test). Source data are provided as a Source Data file. **c:** Schematic of lentiviral vector used for generating HMC3 cells with stable expression of rM3Ds-P2A-EGFP. LTR: long terminal repeat. PGK: phosphoglycerate kinase promoter. Images show maximum intensity projections of HMC3 cells immunostained for the VSV-G tag under non-permeabilizing conditions. Magenta: VSV-G. Green: EGFP. Blue: nuclear staining with Hoechst. **d-e:** RT-qPCR for genes derived from cluster A and C. HMC3 cell lines simultaneously treated with IFNγ/IL1β and combinations of levalbuterol (LB) or CNO. **d:** Principal component analysis based on log2 fold changes compared to IFNγ/IL1β alone in the same cell line. Source data are provided as a Source Data file. **e:** Hierarchical clustering of HMC3 cell samples (columns) and genes (rows). Upregulation (green) and downregulation (blue) compared to IFNγ/IL1β alone in the same cell line. Source data are provided as a Source Data file.

Supplementary Figure S17

In-silico design of CNO-responsive chimeras mimicking a GPCR-of-interest

a

Signal peptide
VSV-G epitope
hM3Dq (ligand binding)
GPCR-of-interest (signaling)

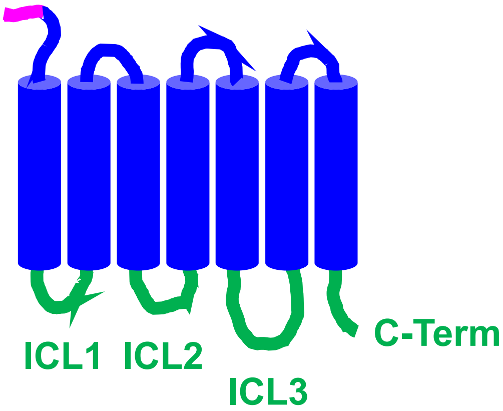

b **hM3Dq ligand binding (protein sequence)**

```
MKTIIALSYIFCLVFAMYTDIEMNRLGKDSLTLHNNSTTSPLFPNISSSWIHSPSDAGLPPGTVTHFGSYNVSRAA
GNFSSPDGTTDDPLGGHTVWQVVFIAFLTGILALVTIIGNILVIVS ICL1 NNYFLLSLACADLIIGVISMNLFT
TYIIMNRWALGNLACDLWLAIDCVASNASVMNLLVISFDRYFSI ICL2 AGVMIGLAWVISFVLWAPAILFWQYF
VGKRTVPPGECFIQFLSEPTITFGTAIAGFYMPVTIMTILYW ICL3 QTLSAILLAFIITWTPYNIMVLVNTFCD
SCIPKTFWNLGYWLCYINSTVNPVCYALCNKTFR C-TERM
```

c **hM3Dq ligand binding (coding DNA sequence)**

```
ATGAAAACGATCATCGCCCTGAGCTACATCTTCTGCCTGGTATTCGCCATGTACACCGATATAGAGATGAACAGGC
TGGGAAAGGATAGCCTCACCTTGCACAATAACAGTACAACCTCGCCTTTGTTTCCAAACATCAGCTCCTCCTGGAT
ACACAGCCCCTCCGATGCAGGGCTGCCCCCGGGAACCGTCACTCATTTCGGCAGCTACAATGTTTCTCGAGCAGCT
GGCAATTTCTCCTCTCCAGACGGTACCACCGATGACCCTCTGGGAGGTCATACCGTCTGGCAAGTGGTCTTCATCG
CTTTCTTAACGGGCATCCTGGCCTTGGTGACCATCATCGGCAACATCCTGGTAATTGTGTCA ICL1 AACAACTA
CTTCCTCTTAAGCCTGGCCTGTGCCGATCTGATTATCGGGGTCATTTCAATGAATCTGTTTACGACCTACATCATC
ATGAATCGATGGGCCTTAGGGAACTTGGCCTGTGACCTCTGGCTTGCCATTGACTGCGTAGCCAGCAATGCCTCTG
TTATGAATCTTCTGGTCATCAGCTTTGACAGATACTTTTCCATC ICL2 GCCGGTGTGATGATCGGTCTGGCTTG
GGTCATCTCCTTTGTCCTTTGGGCTCCTGCCATCTTGTTCTGGCAATACTTTGTTGGAAAGAGAACTGTGCCTCCG
GGAGAGTGCTTCATTCAGTTCCTCAGTGAGCCCACCATTACTTTTGGCACAGCCATCGCTGGTTTTTATATGCCTG
TCACCATTATGACTATTTTATACTGG ICL3 CAGACCCTCAGTGCGATCTTGCTTGCCTTCATCATCACTTGGAC
CCCATACAACATCATGGTTCTGGTGAACACCTTTTGTGACAGCTGCATACCCAAAACCTTTTGGAATCTGGGCTAC
TGGCTGTGCTACATCAACAGCACCGTGAACCCCGTGTGCTATGCTCTGTGCAACAAAACATTCAGA C-TERM
```

Supplementary Figure S17: In-silico design of DREADD-based chimeras for a GPCR-of-interest.

a: Schematic of a DREADD-based GPCR chimera containing signal peptide (black) and VSV-G epitope tag (magenta) at the N-terminus. Blue: ligand binding domains of the DREADD hM3Dq. Green: signaling domains of a GPCR-of-interest. **b-c:** Protein (**b**) and corresponding coding DNA sequence (**c**) of hM3Dq ligand binding domains. To generate a DREADD-based chimera for a specific GPCR-of-interest, sequences encoding for the respective signaling domains (green) are inserted in the designated fields. ICL1-3, intracellular loops 1-3. C-Term, C-terminus. Protein sequences of putative signaling domains of 292 GPCRs-of-interest are available in Supplementary Data 1.

Supplementary Table S1 – HEK cell transfection scheme.

Culture vessel	Transfection mix per well	Total DNA per well	Cell suspension per well	Total cell number per well
**96-well plate**	10µl	200ng	90µl	50,000
**6-well plate**	300µl	6µg	1,700µl	1,500,000
**8-well chamber slide**	30µl	600ng	170µl	150,000

Supplementary Table S2 (continued)

List of RT-qPCR primers								
**ID**	**Species**	**Target transcript**	**Sequence**	**Amplicon size**	**Transcript variants**	**Targeted exons**	**Annealing temperature**	**Efficiency**
OAZ1	Human	NM_004152.3	FW: AGGACAGCTTTGCAGTTCTC<br>RV: CGGTTCTTGTGGAAGCAAATG	82bp	1, 2	4, 5	60°C	87%
GAPDH	Human	NM_002046.7	FW: GTCTCCTCTGACTTCAACAGCG<br>RV: ACCACCCTGTTGCTGTAGCCAA	131bp	1, 2, 3, 4, 7	8, 9	60°C	88%
ACTB	Human	NM_001101.5	FW: CACCATTGGCAATGAGCGGTTC<br>RV: AGGTCTTTGCGGATGTCCACGT	135bp	-; also recognizes ACTG1 transcripts	4, 5	60°C	84%
RPL27	Human	NM_000988.5	FW: ATCGCCAAGAGATCAAAGATAA<br>RV: TCTGAAGACATCCTTATTGACG	123bp	1, 2, 3	Variant 1 and 2: 3, 4; Variant 3: 2, 3	60°C	91%
TNF	Human	NM_000594.4	FW: GCACTTTGGAGTGATCGG<br>RV: TTCGAGAAGATGATCTGACTGC	95bp	-	1, 2, 3	60°C	90%
IL1β	Human	NM_000576.3	FW: ATGATGGCTTATTACAGTGGCAA<br>RV: GTCGGAGATTCGTAGCTGGA	132bp	-	2, 3, 4	60°C	90%
IL6	Human	NM_000600.5	FW: GGCACTGGCAGAAAACAACC<br>RV: GCAAGTCTCCTCATTGAATCC	85bp	1, 2, 3	Variant 1 and 3: 3, 4; Variant 2: 2, 3	60°C	91%
β2AR	Human	NM_000024	Commercially available and validated RT-qPCR primer pair (Integrated DNA Technologies; Hs.PT.56a.23196446.g)	NA	-	NA	60°C	NA
GBP2	Human	NM_004120.5	FW: AAGGAAGGGGATACAGGCCAAA<br>RV: TGCATCAGCCACATCCTCCTTG	70bp	NA	NA	60°C	91%
C17orf100	Human	NM_001105520.2	FW: TTTACTGACCGTCCTGCGTCTT<br>RV: AAGGTTTTCTGAGGGCTGTGGA	182bp	NA	NA	60°C	94%
CBWD3	Human	NM_201453.4	FW: GAAACGGTTGCCTCTGCTGTTC<br>RV: AGGGTCTGCTAATCCAGTGGTC	119bp	NA	NA	60°C	99%
FBXO10	Human	NM_012166.3	FW: AGATGGTGTGGTTGTGGGAGAC<br>RV: ACCACAGCCCTTGTTAGCGTA	76bp	NA	NA	60°C	92%
IFI27	Human	NM_001130080.3	FW: CGTCCTCCATAGCAGCCAAGAT<br>RV: ACCCAATGGAGCCCAGGATGAA	147bp	NA	NA	60°C	97%
PAG1	Human	NM_018440.4	FW: TTCAGCCGTTCAGTTACTAGCC<br>RV: TGGACTTCCTCGTAATGCTGC	131bp	NA	NA	60°C	83%
TFAP2A-AS1	Human	NR_033910.1	FW: CCTCGCAGTCCTCGTACTTGAT<br>RV: AGGCTGTTGGTAAAGAGCCAGA	129bp	NA	NA	60°C	95%
EPHA2	Human	NM_004431.5	FW: ACTGCCAGTGTCAGCATCAACC<br>RV: GTGACCTCGTACTTCCACACTC	131bp	NA	NA	60°C	89%
FLT1	Human	NM_001159920	FW: TGCCGGGTTACGTCACCTA<br>RV: GTCCCAGATTATGCGTTTTCCAT	90bp	NA	NA	60°C	97%
H3F3A	Human	NM_002107.7	FW: ACAAAAGCCGCTCGCAAGAGTG<br>RV: TTTCTCGCACCAGACGCTGGAA	157bp	NA	NA	60°C	86%
LIF	Human	NM_001257135.2	FW: AGGTCTTGGCGGCAGTACAC<br>RV: GAGGTGCCAAGGTACACGACTA	161bp	2	NA	60°C	83%

Supplementary Table S2 (continued)

List of RT-qPCR primers								
LINC01003	Human	NR_027387.1	FW: GTAAAGCCGGATCTGTCCAACG<br>RV: AGCATGGAGAAAAGGGATGGGT	99bp	NA	NA	60°C	104%
SCARF1	Human	NR_028075.3	FW: TGACAGTCTCACATCACGACCC<br>RV: CACACAGTAGGCAGGAACCTCA	140bp	NA	NA	60°C	76%
SLC41A2	Human	NM_032148.6	FW: GCCAAACATCCAGCCACAAGAAC<br>RV: GGGTCTGATACAGTTGTGTCCAG	113bp	NA	NA	60°C	93%
SLIT2	Human	NM_001289136	FW: CAGAGCTTCAGCAACATGACCC<br>RV: GAAAGCACCTTCAGGCACAACAG	153bp	NA	NA	60°C	78%
TGIF2-C20orf24	Human	NM_001199535	FW: AACCTGTCAGTGCTGCAAGATG<br>FW: TCCCAAGAACCCTCGTAATGGC	114bp	NA	NA	60°C	92%

Efficiencies were validated from the slope of four to five serial 1:4 dilutions of cDNA template according to the following formula:

$$Efficiency = (2^{(-\frac{1}{Slope})}) - 1$$
