# Supplementary material for: Chimeric GPCRs mimic distinct signaling pathways and modulate microglia responses
Source: Nat Commun. 2022 Aug 15;13:4728. doi: 10.1038/s41467-022-32390-1 (PMC9378622; doi:10.1038/s41467-022-32390-1)
Supplement: Supplementary file 3 — Description of Additional Supplementary Files [file 41467_2022_32390_MOESM3_ESM.pdf]

Description of Additional Supplementary Files

Supplementary Data 1: Putative GPCR signaling domains of all receptors included in the multiple protein sequence alignment.

Supplementary Data 2: Results of differential gene expression analysis of the mRNA sequencing experiment.

Supplementary Data 3: Details on R environment, statistical models, and test parameters for all graphs.
