# Supplementary material for: Chimeric GPCRs mimic distinct signaling pathways and modulate microglia responses
Source: Nat Commun. 2022 Aug 15;13:4728. doi: 10.1038/s41467-022-32390-1 (PMC9378622; doi:10.1038/s41467-022-32390-1)
Supplement: Supplementary file 7 — Reporting Summary [file 41467_2022_32390_MOESM7_ESM.pdf]

nature portfolio

Corresponding author(s): Sandra Siegert

Last updated by author(s): Jul 6, 2022

Reporting Summary

Nature Portfolio wishes to improve the reproducibility of the work that we publish. This form provides structure for consistency and transparency in reporting. For further information on Nature Portfolio policies, see our Editorial Policies and the Editorial Policy Checklist.

Statistics

For all statistical analyses, confirm that the following items are present in the figure legend, table legend, main text, or Methods section.

n/a	Confirmed	
☐	☒	The exact sample size (*n*) for each experimental group/condition, given as a discrete number and unit of measurement
☐	☒	A statement on whether measurements were taken from distinct samples or whether the same sample was measured repeatedly
☐	☒	The statistical test(s) used AND whether they are one- or two-sided<br>*Only common tests should be described solely by name; describe more complex techniques in the Methods section.*
☐	☒	A description of all covariates tested
☐	☒	A description of any assumptions or corrections, such as tests of normality and adjustment for multiple comparisons
☐	☒	A full description of the statistical parameters including central tendency (e.g. means) or other basic estimates (e.g. regression coefficient) AND variation (e.g. standard deviation) or associated estimates of uncertainty (e.g. confidence intervals)
☐	☒	For null hypothesis testing, the test statistic (e.g. *F*, *t*, *r*) with confidence intervals, effect sizes, degrees of freedom and *P* value noted<br>*Give P values as exact values whenever suitable.*
☒	☐	For Bayesian analysis, information on the choice of priors and Markov chain Monte Carlo settings
☒	☐	For hierarchical and complex designs, identification of the appropriate level for tests and full reporting of outcomes
☐	☒	Estimates of effect sizes (e.g. Cohen's *d*, Pearson's *r*), indicating how they were calculated

Our web collection on statistics for biologists contains articles on many of the points above.

Software and code

Policy information about availability of computer code

Data collection	Information on GPCR sequences collected from IUPHAR/BPS (www.guidetopharmacology.org).<br>Protein crystal structures collected from the PDB data base (www.rcsb.org).
Data analysis	Statistical analysis and data visualization with R (version 4.0.2).<br>mRNA sequencing data analysis with DESeq2 (version 1.28.1).<br>Multiple protein sequence alignment with MUSCLE (MUSCLE does not have a version number; citation is provided in the method section).<br>Alignment visualization and analysis with Jalview (version 2.9.0b2).<br>Transmembrane helix prediction with TMHMM (www.cbs.dtu.dk/services/TMHMM).<br>Signal peptide prediction with SignalP (www.cbs.dtu.dk/services/SignalP).<br>Protein crystal structure visualization with VMD (version 1.9.2).<br>Densitometry analysis of Western blot bands with Bio-Rad Image Lab (version 6.0.1).<br>Analysis of calcium imaging with Peakcaller (https://hussmanautism.org/resources/software) which was run via Matlab 2017a (Peakcaller does not have a version number; citation is provided in the method section).

For manuscripts utilizing custom algorithms or software that are central to the research but not yet described in published literature, software must be made available to editors and reviewers. We strongly encourage code deposition in a community repository (e.g. GitHub). See the Nature Portfolio guidelines for submitting code & software for further information.

March 2021

Data

Policy information about availability of data

All manuscripts must include a data availability statement. This statement should provide the following information, where applicable:

- Accession codes, unique identifiers, or web links for publicly available datasets
- A description of any restrictions on data availability
- For clinical datasets or third party data, please ensure that the statement adheres to our policy

Our mRNA sequencing data is deposited in NCBI's Gene Expression Omnibus and are accessible through GEO Series accession number GSE194125. Source data for each graph are provided with this paper and under doi.org/10.15479/AT:ISTA:11542. Information on GPCR sequences was collected from IUPHAR/BPS (www.guidetopharmacology.org). Protein crystal structures were collected from the PDB data base (www.rcsb.org) for visualization of bovine RHO (PDB ID: 1U19), rat CHRM3 (PDB ID: 4U15) as surrogate for hM3Dq, and human β2AR (PDB ID: 2RH1). Retina transcriptome data is accessible under GSE33089.

Field-specific reporting

Please select the one below that is the best fit for your research. If you are not sure, read the appropriate sections before making your selection.

☒ Life sciences ☐ Behavioural & social sciences ☐ Ecological, evolutionary & environmental sciences

For a reference copy of the document with all sections, see nature.com/documents/nr-reporting-summary-flat.pdf

Life sciences study design

All studies must disclose on these points even when the disclosure is negative.

Sample size	No sample-size calculations were performed. Sample size was estimated based on literature reports of similar experiments (https://doi.org/10.1038/s41467-018-04342-1; https://doi.org/10.1016/j.celrep.2019.05.010)
Data exclusions	No data were excluded.
Replication	Luciferase assays were performed with three technical replicates and at least three independent experimental repetitions. Exact number of repetitions is provided in the respective figure legends for each graph. RT-qPCR was performed with three technical replicates and three to four experimental repetitions. For mRNA sequencing and Western blots three independent samples were included without technical replicates. Representative immunostainings were replicated at least two times. All replication attempts were successful.
Randomization	Not applicable. In-vitro assays were performed by seeding cells from the same culture dish, cell suspension, and cell passage. For primary mouse microglia cultures, all pups from a litter were pooled.
Blinding	No blinding was performed. Experimental procedure and analysis were performed by different persons. Assay readout was automatically generated by the respective devices such as luciferase assay data by a plate reader. mRNA sequencing was carried out at the Vienna BioCenter Core Facility. Image pre-processing was performed with the same parameters for all conditions as described in the methods section.

Reporting for specific materials, systems and methods

We require information from authors about some types of materials, experimental systems and methods used in many studies. Here, indicate whether each material, system or method listed is relevant to your study. If you are not sure if a list item applies to your research, read the appropriate section before selecting a response.

Materials & experimental systems

n/a	Involved in the study	
☐	☒	Antibodies
☐	☒	Eukaryotic cell lines
☒	☐	Palaeontology and archaeology
☐	☒	Animals and other organisms
☒	☐	Human research participants
☒	☐	Clinical data
☒	☐	Dual use research of concern

Methods

n/a	Involved in the study	
☒	☐	ChIP-seq
☒	☐	Flow cytometry
☒	☐	MRI-based neuroimaging

Antibodies

Antibodies used	Mouse anti-VSV-G conjugated with Cy3 (Sigma; C7706; clone P5D4; LOT: 049M4837V).<br>Rabbit-anti-pERK1/2 (Cell Signaling Technology; 9101S; LOT: 30).

	Rabbit-anti-GAPDH (Sigma; ABS16; LOT: 3275069).<br>Donkey-anti-rabbit conjugated with HRP (GE Healthcare; NA934V; LOT: 16976257)
Validation	The same anti-VSV-G antibody clone (P5D4) has been previously used in published articles for detecting VSV-G (doi: 10.1016/j.celrep.2020.108042; doi: 10.7554/eLife.57763). Additionally, the specificity of anti-VSV-G antibody to recognize VSV-G-tagged GPCRs is also demonstrated through negative controls in the manuscript (see Supplementary Fig.S2c-g).<br>Anti-pERK1/2 has been used for Western blot analysis in previous studies (e.g. doi: 10.1038/s41467-020-19877-5).<br>Anti-GAPDH antibody was a gift from the Novarino group at IST who are routinely utilizing this product for Western blot analysis.<br>Additionally, this anti-GAPDH antibody has also been used recent publications (for example doi: 10.1038/s41467-017-02089-9).

Eukaryotic cell lines

Policy information about cell lines

Cell line source(s)	HEK293T cells (CRL-3216) obtained from ATCC.<br>HMC3 cells (CRL-3304) obtained from ATCC.
Authentication	Certified cell lines were purchased from ATCC and matched the morphological description provided by the manufacturer.<br>Human origin of both cell lines has been confirmed by successful PCR amplification of human gene sequences.
Mycoplasma contamination	Mycoplasma tests are routinely performed at ISTA cell culture facilities and came back negative for our cell lines.
Commonly misidentified lines<br>(See ICLAC register)	HEK293T cells (these cells are commonly used for studying GPCRs due to their favorable transfection and culture conditions; see also doi: 10.1186/1471-2164-12-14)

Animals and other organisms

Policy information about studies involving animals; ARRIVE guidelines recommended for reporting animal research

Laboratory animals	C57BL/6J mice (Cat#000664) were purchased from The Jackson Laboratories and housed in the ISTA Preclinical Facility.<br>Newborn litters of mixed sex aged P0-P3 were used for primary microglia cultures.
Wild animals	Study did not involve wild animals.
Field-collected samples	Study did not involve field-collected samples.
Ethics oversight	Animal procedures are approved by the Bundesministerium für Wissenschaft, Forschung und Wirtschaft (bmwfw) Tierversuchsgesetz 2012 (TVG 2012), BGBI. I Nr. 114/2012, idF BGBI. I Nr. 31/2018 under the number GZ 2021-0.262.895.

Note that full information on the approval of the study protocol must also be provided in the manuscript.
